# Effect of Different Dietary Patterns on Cardiometabolic Risk Factors: An Umbrella Review of Systematic Reviews and Meta-Analyses

**DOI:** 10.3390/nu16223873

**Published:** 2024-11-13

**Authors:** Christina A. Chatzi, Athanasios Basios, Georgios Markozannes, Evangelia E. Ntzani, Konstantinos K. Tsilidis, Kyriakos Kazakos, Aris P. Agouridis, Fotios Barkas, Maria Pappa, Niki Katsiki, Evangelos C. Rizos

**Affiliations:** 1 Department of Nursing, School of Health Sciences, University of Ioannina, 45500 Ioannina, Greece; xatzixristina@uoi.gr (C.A.C.);; 2 Laboratory of Hygiene and Epidemiology, Department of Medicine, School of Health Sciences, University of Ioannina, 45500 Ioannina, Greeceentzani@uoi.gr (E.E.N.);; 3 Department of Epidemiology and Biostatistics, School of Public Health, Imperial College London, London SW7 2AZ, UK; 4 Department of Health Services, Policy and Practice, School of Public Health, Brown University, Providence, RI 02912, USA; 5 Department of Nursing, School of Health Sciences, International Hellenic University, 57001 Thessaloniki, Greece; 6 Department of Medicine, School of Medicine, European University Cyprus, 2404 Nicosia, Cyprus; 7 Department of Internal Medicine, German Oncology Center, 4108 Limassol, Cyprus; 8 Department of Medicine, School of Health Sciences, University of Ioannina, 45500 Ioannina, Greece; 9 Department of Rheumatology, Attikon University Hospital, 12462 Athens, Greece; 10 Department of Nutritional Sciences and Dietetics, School of Health Sciences, International Hellenic University, 57400 Thessaloniki, Greece

**Keywords:** cardiometabolic risk factors, dietary pattern, umbrella review

## Abstract

Background/Objectives: Lifestyle interventions such as dietary changes have been proposed to control the cardiometabolic risk factors and thus prevent cardiovascular (CV) disease (CVD). We performed an umbrella review to investigate whether different dietary patterns affect CV risk in individuals with at least one cardiometabolic risk factor (hypertension, dyslipidemia, obesity, diabetes, metabolic syndrome) but not established CVD. Methods: We systematically searched the PubMed and Scopus databases (up to August 2024) for the systematic reviews and meta-analyses of randomized controlled trials (RCTs). Articles should be written in English and refer to a specific dietary pattern (such as Mediterranean diet, etc.). The population studied referred to adults with at least one cardiovascular (CV) risk factor. Results: From 4512 records identified, we finally included 25 meta-analyses with a total of 329 associations. Strong evidence for a benefit was found for LCD with reductions in BW [MD: −4.79 (95% CI −5.85, −3.72) kg, *p* ≤ 0.001], SBP [MD: −6.38 (95% CI −7.84, −4.93) mmHg, *p* ≤ 0.001], TG [WMD: −5.81 (95% CI −7.96, −3.66) mg/dL, *p* ≤ 0.001], and fasting plasma insulin [MD: −15.35 (95% CI −19.58, −11.12) pmol/L, *p* ≤ 0.001], as well as for low-GI diet for the reduction of BW [SMD: −0.66 (95% CI −0.90, −0.43) kg, *p* ≤ 0.001]. Conclusions: Across many dietary patterns, LCD showed strong or highly suggestive evidence for a benefit on SBP, BW reduction, and lipid profile improvement. Secondarily, low-GI, DASH, and Portfolio and Nordic diets suggested beneficial effects on controlling CV risk.

## 1. Introduction

Cardiovascular (CV) disease (CVD), accounts for 17 million deaths every year according to the World Health Organization (WHO) [1]. Among the traditional CV risk factors, dyslipidemia (increased levels of total cholesterol (TC) and low-density lipoprotein cholesterol (LDL-c), decreased levels of high-density lipoprotein cholesterol (HDL-c) and, to a lesser extent, increased triglycerides (TG) levels), hypertension, obesity, metabolic syndrome (MetS), and type 2 diabetes (T2D) are closely related to each other [1,2,3,4,5,6,7].

Lifestyle interventions such as dietary changes, physical activity, smoking cessation and reduction of alcohol consumption have a positive effect on cardiometabolic risk factors, reducing the prevalence of hypertension, hyperlipidemia, obesity, and insulin resistance [1,4,7,8]. Meta-analyses and systematic reviews provide evidence for the adverse effect of unhealthy, non-balanced diets on health status [9,10]. However, factors such as modest effect sizes, limited number of studies, small number of assessed populations, and overall low-quality studies included in the meta-analyses are indications of the possibility of bias (publication or other selective reporting biases). The reported heterogeneity is usually high and is also related to different dietary interventions, a wide range of outcomes, and variety in the duration of diets, thus providing conflicting results for the effect of selected dietary patterns on specific outcomes [9,10]. Umbrella reviews in this field usually include mixed populations (healthy, individuals with CV risk factors, patients with established CVD) and meta-analyses of both observational and RCTs [11,12,13,14,15,16]. To assess the overall effect of various types of diets and diminish the likelihood of these biases, we performed an umbrella review approach of meta-analyses that included RCTs to investigate whether different dietary patterns affect CV risk in individuals with at least one cardiometabolic risk factor but not established CVD.

## 2. Materials and Methods

### 2.1. Literature Search—Data Extraction

We identified all relevant meta-analyses examining the association of different dietary patterns with cardiometabolic risk factors. We systematically searched PubMed and Scopus databases from their inception through October 2024 for systematic reviews and meta-analyses of RCTs. Two authors (C.A.C. and A.B.) separately extracted the data, and any discrepancy was resolved by a third author (G.M.). The Rayyan-AI Powered Tool for systematic literature review was used for screening. The Rayyan-AI Powered Tool is freely available, and the company had no involvement in any stage of our research. To double-check our search, we also used the traditional way of screening. At the meta-analysis level, we abstracted information on first author, year of publication, distinctive population characteristics, examined interventions, outcomes, number of included studies, and meta-analysis metric (risk ratio (RR), odds ratio (OR), or hazard ratio (HR) for dichotomous and mean difference (MD), weighted mean difference (WMD), and standardized mean difference (SMD)) for continuous outcomes. At the individual study level, we abstracted information on first author’s name, year of publication, study design, sample size, information on adjustment factors, number of events or number of cases, effect estimate, and its measure of variation (such as standard error (SE), 95% confidence interval (CI), *p*-value). This umbrella review is in accordance with the Preferred Reporting Items for Overviews of Reviews (PRIOR) statement [17], and the protocol has been registered in the Open Science Framework (OSF) (Registration DOI: https://doi.org/10.17605/OSF.IO/D9J8C, 25 October 2023).

### 2.2. Inclusion/Exclusion Criteria

Articles should be meta-analyses of RCTs, written in English, and refer to a certain dietary pattern (such as Mediterranean diet, LCD, or low-fat diet), but not to an individual food group. The population studied male and female adults (>18 years old) with one or more CV risk factors (such as dyslipidemia, high blood pressure, MetS, T2D, obesity) but not established CVD. Any article assessing medication, studies referring to animals or participants with chronic diseases such as liver failure, chronic kidney disease, human immunodeficiency virus (HIV), alcohol abuse, cancer, or organ transplants were excluded.

### 2.3. Quality Assessment

The online checklist called Assessing the Methodological Quality of Systematic Reviews (AMSTAR 2) was used to assess methodological quality and assign an overall rating for the reviews included [18].

### 2.4. Statistical Analysis

We graded the evidence of each association into 4 classes of evidence (strong, highly suggestive, suggestive, weak) using the established umbrella reviews criteria [19]. Strong evidence considered the associations having >500 cases, *p* value < 10^−6^ by the random-effects model, *I*^2^ < 50%, 95% PI excluding the null, no small-study effects, and no excess significance bias (to evaluate whether there was a relative excess of formally significant findings in the published literature for any reason) [19]. Highly suggestive evidence required number of cases >500, *p* < 10^−6^ by the random-effects model, largest study with a statistically significant effect, and class I criteria were not met. Suggestive evidence required >500 cases, *p* < 10^−3^ by the random-effects model and class I–II criteria were not met. Weak evidence (class IV) was considered when *p* < 0.05 and class I–III criteria were not met. Associations with a *p*-value > 0.05 in the random-effects meta-analysis were considered non-significant [9]. *I*^2^ describes the between-studies variation that can be attributed to heterogeneity rather than sampling error. It lies between 0% and 100%. *I*^2^ values of 25%, 50%, and 75% indicate low, moderate, and large heterogeneity, respectively [10,20,21]. An Egger’s regression asymmetry test *p*-value ≤ 0.1, along with an inflated random effects estimate compared with the point estimate of the largest and most precise study (smallest standard error) in the meta-analysis, was used as an indication of small-study effects bias [21]. The statistical analysis was performed with Statistics and Data (STATA) version 16.

## 3. Results

### 3.1. Main Results

**Figure 1:**
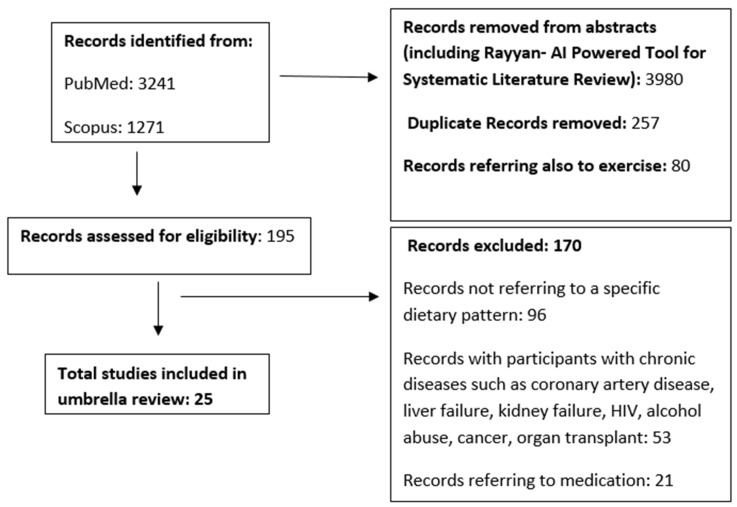
Flow chart of the umbrella review.

In total, from 4512 records identified, we finally included 25 meta-analyses (Figure 1). Three of them assessed low-GI diet (population: individuals with T2D or type 1 diabetes (T1D) or gestational diabetes), five ketogenic diet (population: overweight/obese individuals with or without T2D, patients with T2D, or at least one cardiometabolic risk factor), five LCD (population: obese or T2D individuals), two Nordic diet (population: individuals with diabetes or at risk of diabetes, or individuals with at least one cardiometabolic risk factor), two vegan/vegetarian diet (population: overweight, or individuals with T2D/prediabetes, or individuals with at least one cardiometabolic risk factor), two Mediterranean diet (population: individuals with T2D), two HP diet (population: individuals with or without diabetes), one Portfolio dietary pattern (population: individuals with hyperlipidemia), one DASH diet (population: Individuals with at least one cardiometabolic risk factor), one comparing Mediterranean, LCD, low-GI, and HP diet (population: individuals with T2D) and one comparing Mediterranean, LCD, DASH, and Nordic diet. Twenty-two articles included exclusively RCTs, whereas the remaining three [22,23,24] included a mixed methodology of randomized and non-randomized articles in the systematic reviews. Overall, 26 outcomes were studied, with the most common being TC, TG, HDL-C, LDL-C (19 reviews), BW or BMI (16 reviews), SBP and DBP (15 reviews), HbA1c (14 reviews), as well as FPG or FPI (11 reviews). The included 25 meta-analyses had a total of 329 associations as each outcome is studied in more than one article. The male/ female ratio could not be estimated since the partial ratio is missing in 20 out of 25 meta-analyses. Study characteristics can be found in Table 1; Table 2 describes each dietary pattern and the overall outcomes, whereas Table 3. summarizes strong and highly suggestive evidence. The results (Supplementary Table S1) are categorized based on the dietary pattern they examine (Mediterranean, LCD, low-GI, ketogenic, HP, DASH, Portfolio, Nordic, vegetarian) and presented only if they offer strong, highly suggestive, or suggestive evidence. When the results are strong, highly suggestive, or suggestive for an outcome that is reported more than once, we report the result with the highest magnitude of effect. The overall quality assessment rated 7 articles as “Low”, whereas the remaining 18 were rated as “Critically Low” (Table 1).

#### 3.1.1. Mediterranean Diet

Fourteen associations referred to the Mediterranean diet. Overall, none of the associations were claimed to be strong or highly suggestive. Three associations referring to HbA1c [MD: −0.30 (95% CI −0.46, −0.14) %, *p* value ≤ 0.001], BMI [MD: −0.29 (95% CI −0.45, −0.13) kg/m^2^, *p* value = 0.001], and FPI [MD: −9.91 (95% CI −14.53, −5.29) pmol/L, *p* value ≤ 0.001] were claimed as suggestive, whereas eleven associations were categorized as weaker or non-significant, all referring to adult males and females with T2D or at least one cardiometabolic risk factor.

#### 3.1.2. Low-Carbohydrate Diet—LCD

In total, 82 associations were assessed for LCD. We found 4 associations referring to BW [MD: −4.79 (95% CI −5.85, −3.72) kg, *p* value ≤ 0.001], SBP [MD: −6.38 (95% CI −7.84, −4.93) mmHg, *p* value ≤ 0.001], FPI [MD: −15.35 (95% CI −19.58, −11.12) pmol/L, *p* value ≤ 0,001] in obese individuals, and TG [WMD: −5.81 (95% CI −7.96, −3.66) mg/dL, *p* value ≤ 0.001] in individuals with T2D, which were claimed as strong; 13 associations referring to BMI [MD: −2.03 (95% CI −2.62, −1.45) kg/m^2,^, *p* value ≤ 0.001], BW [MD: −7.44 (95% CI −9.07, −5.81) kg, *p* value ≤ 0.001], waist circumference (WC) [MD: −6.58 (95% CI −8.14, −5.02) cm, *p* value ≤ 0.001], HDL-c [MD: 6.71 (95% CI 4.80, 8.61) mg/dL, *p* value ≤ 0.001], TG [MD: −38.85 (95% CI −48.27, −29.43) mg/dL, *p* value ≤ 0.001], SBP [MD: −5.54 (95% CI −7.50, −3.57) mmHg, *p* value ≤ 0.001], and DBP [MD: −3.96 (95% CI −5.31, −2.60) mmHg, *p* value ≤ 0.001] in obese males and females as highly suggestive; 10 associations referring to BMI [MD: −1.41 (95% CI −2.15, −0.66) kg/m^2^, *p* value ≤ 0.001] and HbA1c [WMD: −0.43 (95% CI −0.63, −0.24) %, *p* value ≤ 0.001 ] in patients with T2D, WC [MD: −6.79 (95% CI −9.94, −3.64) cm, *p* value ≤ 0.001], HDL-C [MD: 2.24 (95% CI 1.11, 3.36) mg/dL, *p* value ≤ 0.001], TG [MD: −23.19 (95% CI −35.54, −10.84) mg/dL, *p* value ≤ 0.001], FPG [MD: −4.00 (95% CI −6.38, −1.62) mg/dL, *p* value = 0.001], SBP [MD: −4.12 (95% CI −6.11, −2.13) mmHg, *p* value ≤ 0.001], and DBP [MD: −2.93 (95% CI −4.35, −1.50) mmHg, *p* value ≤ 0.001] in obese individuals, which were claimed as suggestive; whereas 55 associations referring to overweight/obese individuals, individuals with T1D/T2D, or presenting with at least one other cardiometabolic risk factor were categorized as weak or non-significant.

#### 3.1.3. Low-Glycemic Index (GI) Diet

We found 29 associations referring to low-GI diets. Only one association referring to BW [SMD: −0.66 (95% CI −0.90, −0.43) kg, *p* value ≤ 0.001] was claimed as strong; one association referring to FPG [SMD: −5.86 (95% CI −8.10, −3.62) mg/dL, *p* value ≤ 0.001] as highly suggestive; and two associations referring to HbA1c [SMD: −0.32 (95% CI −0.45, −0.19)%, *p* value ≤ 0.001] and LDL-c [−3.32 (95% CI −4.98, −1.66) mg/dL, *p* value ≤ 0.001], referring to adult individuals with T1D or T2D as suggestive. There were 25 associations referring to individuals with T1D or T2D that were categorized as weak or non-significant.

#### 3.1.4. Ketogenic Diet

Overall, 121 associations referred to the ketogenic diet. No association was claimed as strong or highly suggestive. Only 1 association referring to TG in patients with T2D [SMD: −0.42 (95% CI −0.64, −0.19) mg/dL, *p* value ≤ 0.001] was claimed as suggestive, whereas 120 associations referring to patients with T2D, or overweight/obese individuals with or without T2D, or with at least one cardiometabolic risk factor, were categorized as weak or non-significant.

#### 3.1.5. High-Protein Diet

Sixteen associations referred to the HP diet. We did not find any association claimed as strong, highly suggestive, or suggestive. All associations referring to individuals with or without T1D or T2D were categorized as weak or non-significant.

#### 3.1.6. DASH Diet

Sixteen associations referred to the DASH diet. No association was claimed as strong, one association referring to SBP [MD: −3.94 (95% CI −5.24, −2.64) mmHg, *p* value ≤ 0.01] was claimed as highly suggestive, and four associations referring to DBP [MD: −2.44 (95% CI −3.44, −1.45) mmHg, *p* value ≤ 0.01], BW [MD: −1.59 (95% CI −2.27, −0.90) kg, *p* value ≤ 0.01], BMI [MD: −0.63 (95% CI −0.92, −0.35) kg/m^2^, *p* value ≤ 0.01], and WC [MD: −1.93 (95% CI −2.80, −1.07) cm, *p* value ≤ 0.01] as suggestive, whereas eleven associations were categorized as weak or non-significant. All associations refer to individuals with at least one cardiometabolic risk factor.

#### 3.1.7. Portfolio Dietary Pattern

Eleven associations referred to the Portfolio dietary pattern. We did not find any association claimed as strong; three associations referring to apolipoprotein B (Apo B) [SMD: −18.13 (95% CI −22.74, −13.51) mg/dL, *p* value ≤ 0.001], LDL-c [SMD: −13.05 (95% CI −16.04, −10.06) mg/dL, *p* value ≤ 0.001], and non-HDL-c [SMD: −14.99 (95% CI −18.43, −11.55) mg/dL, *p* value ≤ 0.001] were claimed as highly suggestive, two associations referring to TG [SMD: −5.04 (95% CI −7.51, −2.58) mg/dL, *p* value ≤ 0.001] and TC [SMD: −13.64 (95% CI −19.94, −7.33) mg/dL, *p* value ≤ 0.001] as suggestive, and six associations were categorized as weak or non-significant. All associations assessed adult patients with dyslipidemia. Of note, all associations derive from a meta-analysis which, besides RCTs, included non-RCTs articles.

#### 3.1.8. Nordic Diet

Twenty-three associations referred to the Nordic diet. No association was claimed as strong, three associations referring to stroke incidence [MD: 0.87 (95% CI 0.78, 0.96)%, *p* value ≤ 0.001], CV mortality [MD: 0.80 (95% CI 0.70, 0.90)%, *p* value ≤ 0.001] and T2D [MD: 0.95 (95% CI 0.85, 1.05)%, *p* value ≤ 0.001] were claimed as highly suggestive, and two associations referring to CVD [MD: 0.80 (95% CI 0.59, 1.02)%, *p* value ≤ 0.001] and coronary heart diseases (CHD) incidence [MD: 0.83 (95% CI 0.64, 1.02)%, *p* value ≤ 0.001] referring to individuals with diabetes or at risk for diabetes were claimed as suggestive. Of note, all these associations were derived from a meta-analysis which, besides RCTs, included non-RCTs articles. Eighteen associations referring to individuals with diabetes, or at risk for diabetes, or at least one cardiometabolic risk factor, were categorized as weak or non-significant.

#### 3.1.9. Vegetarian/Vegan Diet

In total 17 associations were found for a vegetarian diet. We did not find any association claimed as strong, one association referring to HDL-c [WMD: −1.84 (95% CI −2.41, −1.28) mg/dL, *p* value ≤ 0.001] claimed as highly suggestive, and one association referring to TC [WMD: −6.64 (95% CI −9.96, −3.33) mg/dL, *p* value ≤ 0.001] as suggestive both in individuals with at least one cardiometabolic risk factor; meanwhile 15 associations referring to overweight or individuals with T2D or prediabetes or at least one cardiometabolic risk factor were categorized as weak or non-significant.

## 4. Discussion

We found that specific types of diet such as LCD exert a beneficial effect on blood pressure (BP) levels, BW and lipid profile, and low-GI diet induces weight reduction and improves fasting glucose levels. Probable benefits of BP reduction are noticed from the DASH diet, LDL-c reduction from the Portfolio diet, and CV events from the Nordic diet. In contrast, other extremely popular diets such as ketogenic, HP, Portfolio, and vegetarian, which are believed to be beneficial across a variety of CV risk factors, failed to show any strong or even highly suggestive evidence for a clear benefit.

### 4.1. Mediterranean Diet

The Mediterranean diet is based on dietary patterns followed by people living around the Mediterranean Sea. It is characterized by high consumption of monounsaturated fatty acids based on olive oil, high consumption of polyphenols deriving from everyday consumption of fruits, vegetables, whole grain cereals, legumes and low-fat dairy products, and moderate alcohol consumption mostly with meals, as well as weekly consumption of fish, poultry, nuts, and a twice a month consumption of red meat [4].

We found only suggestive evidence that the Mediterranean diet lowers BMI (approximately −30%), HbA1c (approximately −30%), and FPI levels (approximately −10 pmol/L. Previous reports show BW and thus BMI reduction in patients following a diet high in monounsaturated fatty acids such as the Mediterranean diet [6,42]. Moreover, reductions in HbA1c and FPI are attributed to the antioxidant and anti-inflammatory properties of the Mediterranean diet due to consumption of polyphenols and fibers included specifically in olives, fruits, vegetables, whole grains, fish, and red wine [4,7]. It should be noted that adherence to the Mediterranean diet is linked to reduced risk for developing MetS, non-alcoholic fatty liver disease, or CVD [43,44,45,46,47], as well as decreased systemic inflammation [16,30]. Naturally, the effectiveness and acceptability of the Mediterranean diet interventions in non-Mediterranean countries is of concern.

### 4.2. Low-Carbohydrate Diet—LCD

LCD is based on low carbohydrate (especially refined) intake and restrictions to high GI carbohydrates, with a carbohydrate consumption range of ≤15% to <40% per daily intake [2,27,34,41,44,45,46,48].

We found strong evidence that LCD lowers SBP (with a clinically meaningful reduction of 6 mmHg), TG (by 6 mg/dL), BW (by 5 kg), and improves insulin resistance. Highly suggestive evidence shows improvement in lipid profile, BP, and BMI and WC reduction. Our findings are in accordance with the latest guidelines for CV risk reduction, where LCD is reported to reduce BW by 2–3 kg and TG by 23 mg/dL, with an increase in HDL-c by 5 mg/dL [35]. In contrast, previous reports fail to show a benefit on weight management in T2D individuals when LCD is compared with higher-carbohydrate diets, HP, Mediterranean, vegetarian, low-GI diets [12].

### 4.3. Low-Glycemic Index (GI) Diet

GI is a measure of the postprandial glycemic response to carbohydrate consumption and is expressed in comparison with a reference food (commonly glucose or white bread) [40]. A GI of ≤55 is considered low, 56–69 is medium, and ≥70 is high based on a glucose scale [30].

We found strong evidence for an improvement in BW and highly suggestive evidence for a decrease in FPG levels, which explains why low-GI diets are so popular for glucose control in people with diabetes [30,40]. Similar reductions in fasting glucose levels are previously reported [30,42] and are attributed to the reduction of hyperglycemia, hyperinsulinemia, and free fatty acids levels, resulting to suppression of the inflammatory response [5].

### 4.4. Ketogenic Diet

The ketogenic diet is characterized by low carbohydrate consumption and moderate protein intake, whereas fat intake is unrestricted. Usually, carbohydrates and protein account for 10% and 20% of daily intake, respectively, and fat consumption approximately for 70% [25,26]. The ketogenic diet is one of the dietary interventions employed by individuals to achieve rapid weight loss, but with a concomitant reduction in muscle mass (although the opposite was initially believed due to the protective effect of ketones on muscle tissue and the increased growth hormone secretion stimulated by low blood glucose, which results in an increase in muscle protein synthesis) [49].

Although we examined 114 associations, we failed to find any association claimed as strong or highly suggestive. Likewise, previous publications show controversial findings that might be explained by the high-fat nature of ketogenic diets, which are often characterized by high cholesterol intake [50]. The latest umbrella review reports a decrease in TG levels but an increase in LDL-c levels [14].

### 4.5. High-Protein Diet

HP diets are characterized by a protein intake of 1.2–1.6 g per kg of body weight per day [8].

Although HP diets are popular for improving muscle mass, enhancing weight loss, glucose control, and reducing CV risk [6,8,51], we failed to show any strong, highly suggestive or even suggestive evidence for a specific benefit. Of note, the avoidance of excess fat and sugar are previously related to unfavorable cardiometabolic status [8].

### 4.6. DASH Diet

The DASH diet was described in the 1990s and consists of 7 servings of carbohydrates, 2 servings of low-fat dairy products, ≤2 servings of lean red meat, 5 servings of vegetables, and 5 servings of fruits daily, with 2 or 3 servings of nuts and seeds per week [1].

Although we failed to show any strong evidence for a specific benefit, the reduction in SBP levels was claimed as highly suggestive. Similarly, in a recent overview, the DASH diet reduces SBP by 9 mmHg, whereas the latest umbrella review shows reductions in both SBP and DBP, related to the consumption of specific components such as fruits and vegetables, whole grains, legumes and pulses, nuts and seeds, total red meat, and poultry intake [11]. The latest European Guidelines for CVD prevention recommend the DASH diet as a beneficial lifestyle intervention for BP reduction [44,45,52,53,54]. In this context, the DASH diet is recommended for an overall improvement in CV risk prevention and management [46]. The increased fiber intake of the DASH diet delays gastric emptying and thus improves serum lipids levels by decreasing macronutrient absorption. This also results in an improvement in insulin sensitivity as well as BP decrease due to insulin association with sodium retention [1].

### 4.7. Portfolio Dietary Pattern

Known as the dietary portfolio or Portfolio diet, it was introduced in the early 2000s as a plant-based dietary pattern. It consists of 42 g of tree nuts or peanuts, 50 g of plant protein (soy products or dietary pulses such as beans and peas), 20 g of viscous soluble fiber (oats, barley, eggplant, apples, oranges, berries) and 2 g of plant sterols from a plant sterol-enriched margarine [22].

We failed to show any strong evidence for a specific benefit, and an improvement in lipid profile was the only benefit claimed as highly suggestive. A similar improvement in lipid profile was previously reported with reductions in LDL-c by 13 mg/dL, TC by 15 mg/dL, and TG by 5 mg/dL [48]. The high content of fibers, sterols, and plant proteins of the Portfolio diet are the components believed to favorably affect the lipid profile (non-HDL-c, ApoB, TC and TG), the inflammatory markers (C-reactive protein (CRP)), and blood pressure levels [22,55,56].

### 4.8. Nordic Diet

Also known as the Baltic Sea diet, the Nordic diet is developed in the Nordic or Northern European region and is characterized by the consumption of whole grain cereals, fruits (mostly berries but also apples and pears), vegetables, legumes (such as oat and barley), rapeseed oil, fatty fish (such as salmon), shellfish, seaweed, low-fat meat choices (such as poultry and game), low-fat dairy products, and the restriction of salt and sugar [33].

We failed to show any strong evidence for a specific benefit, whereas highly suggestive evidence showed a benefit on atherosclerosis-related outcomes. The European Food Safety Authority (EFSA), as well as the latest European Society of Cardiology (ESC) guidelines, also emphasize the benefits on CV risk by the consumption of long chain omega-3 fatty acids [particularly eicosapentaenoic acid (EPA)] included in fatty fish. Although the diet appears to be more beneficial among individuals with MetS or increased BP, it is important to notice that almost every study with the Nordic diet is coupled with BW reduction, which by itself exerts benefits in many CV risk factors such as SBP/DBP levels [24,33,57].

### 4.9. Vegetarian Diet

Vegetarian diet excludes all animal flesh and differentiates to vegan diets (exclude all foods from animal sources), raw vegan, ovovegetarian, lactovegetarian, lacto-ovovegetarian, and pescovegetarian. Vegetarian diets are rich in fibers, magnesium, folic acid, vitamins A and E, omega-6 polyunsaturated fatty acids, and antioxidants but low in total fat and saturated fatty acids, EPA, sodium, zinc, and vitamin B12 [3,29].

We did not find strong evidence for any benefit. Traditionally, high consumption of fruits, vegetables, and generally dietary fiber is commonly recommended for controlling BW and improving the lipid profile, as well as for CV risk reduction [49,52]. The main argument for a potential benefit is related to the high content of phytosterols and flavonoids that characterizes this dietary pattern. Phytosterols compete with cholesterol for a place in the micelles and thus reduce intestinal cholesterol absorption. Flavonoids and saponins also disrupt cholesterol micelle solubility and inhibit LDL-C oxidation, which is also beneficial for CV health [3]. The latest umbrella review [15] shows a potential benefit on cerebrovascular disease, which is hampered by the limited number of studies and moderate overall quality of evidence.

## 5. Conclusions

Dietary patterns vary, and most of them reflect different cultures, everyday needs, and current trends of modern lifestyle. In the present umbrella review, we found strong or highly suggestive evidence for a benefit on BP, BW reduction, as well as improvement lipid profile. Moreover, low-GI, DASH, Portfolio, and Nordic diets suggest beneficial effects for controlling the traditional CV risk factors and reducing atherosclerosis-related events.

## Figures and Tables

**Table 1:** Characteristics of the included meta-analyses.

Author, Year	Country/Region	Design of Included Studies	Intervention	Comparison	Outcomes	Population	Total Sample (*N*)	Age (Years)	Duration/Follow-Up	Quality Assessment AMSTAR 2
Amini et al. (2024) [25]	USA, Israel, Swede, Australia, New Zealand, Canada, Italy, Netherlands, Spain, Norway, Japan	RCTs	Ketogenic diet (≥45% daily intake from fat and ≤50g carbohydrate daily intake)	Any diet	SBP DBP	Adult males and females with at least one cardiovascular risk factor	1664 M: ΝA F: ΝA	25–63 (mean age)	-	Low
Choy and Louie (2023) [26]	Australia, USA, UK, South Africa, Spain, Israel, Germany	RCTs	Ketogenic diet	Any diet	TC, HDL-C, LDL-C, TG HbA1c, FPG BW, BMI, WC, FPI, HOMA SBP, DBP	Adult males and females with T2D	541 M: ΝA F: ΝA	51–65 (mean ages)	-/3–52 weeks	Critically low
Luo et al. (2022) [27]	USA, Norway, Greece, Spain, Italy, Columbia, China, UK, Serbia	RCTs	Ketogenic diet	Non-HP diet	TC, HDL-C, LDL-C, TG BW, BMI WC, Body Fat Volume HbA1c, FPG, FPI, HOMA SBP, DBP Uric acid Creatinine	Adult males and females overweight or obese with or without T2D	1074 M: ΝA F: ΝA	21–65 (mean ages)	-/0.6–48 weeks	Low
Massara et al. (2022) [24]	Denmark, Sweden, Iceland, Finland	RCTs or prospective cohort studies	Nordic diet	Usual diet	CV mortality CVD/CHD/Stroke incidence HDL-C, LDL-C, Non-HDL-C, TG, Apo B WC, BW, BMI HbA1c, FPG, FPI, SBP, DBP CRP	Adult males and females: a. diabetes, no CVD b. no diabetes c. diabetes or risk factors for diabetes	1774 M: ΝA F: ΝA	49–57 (prospective); 48–54 (RCTs)	-/14–18 years (prospective), 12–48 weeks (RCTs)	Critically low
Rafiullah et al. (2022) [28]	USA, Spain, Australia, Israel	RCTs	LCD	Usual diet	HbA1c, BW, TG, LDL-c	Adult males and females with T2D	797 M: 352 F: 445	53–99.7 (mean ages)	3–24 months/-	Low
Termannsen et al. (2022) [29]	USA, Canada, Korea, New Zealand	RCTs	Vegan (no foods from animal sources) diet	Omnivorous diet	TC, HDL-C, LDL-C, TG BW, BMI HbA1c SBP, DBP	Adult males and females overweight or T2D, or prediabetes	1510 M: 386 F: 1124	48–61 (mean ages)	12–26 weeks/-	Critically low
Chiavaroli et al. (2021) [30]	Canada, Australia, France, USA, Israel, Mexico, and other European and Asian countries	RCTs	Low-GI diet	Usual diet	HDL-C, LDL-C, Non-HDL-C, TG, Apo B BW, WC, BMI SBP, DBP FPI, HbA1c, FPG, CRP	Adult males and females with T2D or T1D	1617 M: ΝA F: ΝA-	54–59 (mean ages)	-	Low
Espinoza-Lopez et al. (2021) [31]	Spain, UK, Australia, Germany, Norway	RCTs	Ketogenic diet	Low energy or LFD	BMI, TG, TC, HDL-c, LDL-c	Adult males and females obese	943 M: ΝA F: ΝA	43–60 (mean ages)	4–24 months/-	Critically low
Lari et al. (2021) [1]	USA, Poland, China, Pakistan, Mexico, Greece, Iran, Australia, Croatia, Korea, Iran, Brazil	RCTs	DASH diet	Other dietary patterns	TC, HDL-C, LDL-C, VLDL-C, TG BW, BMI, WC FPG, FPI,, HOMA SBP, DBP CRP	Adult males and females, with at least one cardiovascular risk factor	9488 M: NA F: NA	23–65 (mean ages)	2–52 weeks/-	Low
Smith et al. (2020) [32]	NA	RCTs	Ketogenic diet	Low energy or LFD	BW	Adult males and females with at least one cardiometabolic risk factor	3340 M: ΝA F: ΝA	-	3–24 months/3–24 months	Critically low
Ojo et al. (2019) [5]	Brazil, Canada, China, USA, Malaysia, Greece	RCTs	Low-GI diet	High-GI diet	TC, HDL-C, LDL-C, TG	Adult T2D males and females OR females with gestational diabetes	782 M: NA F: NA	30–63 (mean ages)	-	Critically low
Ramezani-Jolfaie et al. (2019) [33]	Europe	RCTs	Nordic diet	Usual diet	TC, LDL-C, HDL-C, TG SBP, DBP	Adult males and females with at least one cardiovascular risk factor	513 M: ΝA F: ΝA	39–60 (mean ages)	2 weeks–6 months/-	Low
Chiavaroli et al. (2018) [22]	Canada	Randomized or non-randomized controlled trials	Portfolio dietary pattern	Any dietary pattern not providing components of the Portfolio diet	CHD risk TC, HDL-C, LDL-C, Non-HDL-C, TG, Apo B BW SBP, DBP CRP	Adult males and females, with hyperlipidemia	439 M: 192 F: 247	55–65 (mean ages)	-/2–24 weeks	Critically low
Sainsbury et al. (2018) [34]	UK, USA, Australia, Sweden, Israel, Japan, New Zealand, Czech Republic, Austria, Canada	RCTs	LCD	HCD	HbA1c	Adult males and females withT1D or T2D	2405 M: ΝA F: ΝA	38–54	-/3–24 months	Critically low
Zhao et al. (2018) [6]	Australia, USA, New Zealand, Greece, Sweden, UK	RCTs	HP diet	Low-protein diet	TC, HDL-C, LDL-C, TG BW, BMI Fat Mass, Free-fat Mass HbA1c FPG, FPI, SBP, DBP	Adult males and females with diabetes	1099 M: ΝA F: ΝA	47–64 (mean ages)	2–24 weeks/-	Critically low
Meng et al. (2017) [35]	Australia, USA, Sweden, UK, Israel, Japan	RCTs	LCD (≤26% daily carbohydrate intake) diet	Usual diet	TC, HDL-C, LDL-C, TG BW HbA1c, FPG	Adult T2D males and females	734 M: ΝA F: ΝA	-	-/3–24 months	Critically low
Ndanuko et al. (2016) [36]	USA, Italy, Brazil, Iceland, Sweden, Denmark, Finland, Australia, France, Spain, Iran, Germany	RCTs	Mediterranean diet LCD DASH Nordic diet	Usual diet	SBP, DBP, HbA1c	Adult males and females with at least one cardiometabolic risk factor	1957 M: ΝA F: ΝA	18–80	-	Critically low
Nissensohn et al. (2016) [37]	Italy, Israel, Spain, North America	RCTs	Mediterranean diet	Usual diet	SBP, DBP	Adult males and females with at least one cardiometabolic risk factor	7987 Μ: ΝA F: ΝA	20–80	24–48 months/-	Critically low
Wang et al. (2015) [3]	USA, Finland, Sweden, Czech Republic, Australia	RCTs	Vegetarian diet	Omnivorous diet	TC, LDL-C	Adult males and females with at least one cardiovascular risk factor	832 M: 200 F: 632	28–56 (mean age)	3 weeks–19 months/-	Critically low
Huo et al. (2015) [7]	USA, Greece, Israel, Italy, Spain, Australia	RCTs	Mediterranean diet	Usual diet	HDL-C, LDL-C, TC, TG BMI, BW HbA1c, FPI	Adult T2D males and females	1178 M: ΝA F: ΝA	26–77 (range)	4 weeks–4 years/-	Critically low
Ajala et al. (2013) [23]	ΝA	RCTs and systematic reviews	Mediterranean diet, LCD, Low-GI diet, and High-protein diet	Other dietary patterns	HbA1c	Adult T2D males and females	3073 M: ΝA F: ΝA	-	6 months–4 years/-	Critically low
Bueno et al. (2013) [38]	Australia, Israel, USA, UK, New Zealand	RCTs	LCD	LFD	BW, TAG, HDL-c, LDL-c, SBP, DBP	Adult males and females overweight or obese	1577 M: ΝA F: ΝA	39–53 (mean ages)	12–24 months/-	Critically low
Schwingshackl and Hoffmann (2013) [39]	ΝA	RCTs	HP diet	Low-protein diet	HDL-C FPI	Adult males and females with or without T2D	1990 M: ΝA F: ΝA	35–60 (mean ages)	12–24 months/-	Low
Fleming and Codwin (2013) [40]	ΝA	RCTs	Low-GI diet	High-GI diet	TC, LDL-C, TG	Adult males and females with or without diabetes	224 M: 51 F: 173	18–60 (range)	-	Critically low
Santos et al. (2012) [41]	ΝA	RCTs	LCD (≤26% daily carbohydrate intake) diet	Usual diet	HDL-C, LDL-C, TG SBP, DBP BW, BMI, WC FPI, HbA1c, FPG, CRP	Adult obese males and females	3647 M: ΝA F: ΝA	-	-/12 weeks–12 months	Critically low

USA: United States of America; UK: United Kingdom; Apo B: apolipoprotein B; BMI: body mass index; BW: body weight; CRP: C-reactive protein; DBP: diastolic blood pressure; HbA1c: glycated hemoglobin A1c; FPG: fasting plasma glucose; HDL-C: high-density lipoprotein cholesterol; HOMA: homeostatic model assessment; LDL-C: low-density lipoprotein cholesterol; SBP: systolic blood pressure; TC: total cholesterol; TG: triglycerides; T2D: type 2 diabetes; WC: waist circumference, NA: Not available.

**Table 2:** Main characteristics of each dietary pattern alongside their overall outcomes.

Dietary Pattern	Dietary Pattern Food Characteristics	Overall Outcomes
Mediterranean Diet	Olives, olive oil, fruits, vegetables, whole grain cereals, legumes, fish, poultry, low-fat dairy products nuts, wine and red meat	HDL-c, LDL-c, TC, TG, BMI, BW, HbA1c, FPI
LCD	Low carbohydrate (especially refined) intake	HDL-c, LDL-c, TC, TG, BW, HbA1c, FPG, SBP, DBP, BMI, WC, FPI, CRP
Low-GI Diet	Meat, vegetables, legumes, grain cereals, dairy products and most fruits	HDL-c, LDL-c, non-HDL-c, TG, Apo B, BW, WC, BMI, SBP, DBP, FPI, HbA1c, FPG, CRP, TC
Ketogenic Diet	Tuna, sardine, prawns, shrimps, lobster, salmon, kababs, sausages, minced, ham, chicken, eggs, full-fat cheese, mozzarella cheese, cheddar cheese, non-starchy and green-leafy vegetables (e.g., spinach, watercress, eggplant, parsley, mulberry, coriander, mint, artichoke, okra, cabbage, mushroom, avocado, leek, carrot, radish, celery, cauliflower, green pepper, lettuce, cucumber, tomato, olives, lemon, strawberry, avocado, berries	SBP, DBP, TC, HDL-c, LDL-c, TG, HbA11c, FPG, BW, BMI, WC, FPI, HOMA, Body Fat Volume, Uric Acid, Creatinine
HP Diet	Daily protein (animal and plant origin) intake of 1.2–1.6 g per kg of body weight	TC, HCL-c, LDL-c, TG, BW, BMI, HbA1c, FPG, FPI, SBP, DBP, Fat Mass, Free Fat Mass
DASH DIET	Carbohydrates, low-fat dairy products, lean red meat, vegetables, fruits, nuts, and seeds	TC, HDL-c, LDL-c, VLDL-c, TG, BW, BMI, WC, FPG, FPI, HOMA, SBP, DBP, CRP
Portfolio Diet	Tree nuts or peanuts, plant protein (soy products or dietary pulses such as beans and peas), viscous soluble fiber (oats, barley, eggplant, apples, oranges, berries), plant sterols from a plant sterol-enriched margarine	TC, HDL-c, LDL-c, non-HDL-c, TG, Apo B, BW, SBP, DBP, CRP, CHD risk
Nordic Diet	Whole grain cereals, fruits (mostly berries but also apples and pears), vegetables, legumes (such as oat and barley), rapeseed oil, fatty fish (such as salmon), shellfish, seaweed, low-fat meat choices (such as poultry and game), low-fat dairy products and restriction of salt and sugar	HDL-c, LDL- c, non-HDL-c, TC, TG, Apo B, WC, BV, BMI, HbA1c, FPG, FPI, SBP, DBP, CRP, CV mortality, CVD/CHD/stroke incidence
Vegetarian Diet	Excludes all animal flesh and/or animal products. Rich in fibers, magnesium, folic acid, vitamins A and E, omega-6 polyunsaturated fatty acids and antioxidants, but low in total fat and saturated fatty acids, EPA, omega-3 polyunsaturated fatty acids, sodium, zinc, and vitamin B12	TC, HDL-c, LDL-c, TG, BW, BMI, HbA1c, SBP, DBP

**Table 3:** Results for strong and highly suggestive evidence according to diet type.

Author	Year	Intervention	Outcome	Meta-Analysis Metric	Random Effect	Prediction Intervals	Egger’s Test
Strong evidence (grade 500)							
Chiavaroli et al. [30]	2021	Low-GI diet	BW (T2D)	SMD	−0.66 (−0.90, −0.43)	−0.91, −0.41	0.709
Meng et al. [35]	2017	LCD (≤26% daily carbohydrate intake)	TG	WMD	−5.81 (−7.96, −3.66)	−8.40, −3.21	0.540
Santos et al. [41]	2012	LCD (≤26% daily carbohydrate intake)	BW, 24 months	MD	−4.79 (−5.85, −3.72)	−8.57, −1.00	0.709
Santos et al. [41]	2012	LCD (≤26% daily carbohydrate intake)	SBP, 6 months	MD	−6.38 (−7.84, −4.93)	−10.04, −2.73	0.018
Santos et al. [41]	2012	LCD (≤26% daily carbohydrate intake)	FPI, 6–11 months	MD	−15.35 (−19.58, −11.12)	−24.64, −6.06	0.597
Highly suggestive evidence (grade 500)							
Chiavaroli at al. [30]	2021	Low-GI diet	Glucose (T2D)	SMD	−5.86 (−8.10, −3.62)	−12.48, 0.76	0.004
Santos et al. [41]	2012	LCD (≤26% daily carbohydrate intake)	BW, 6 months	MD	−5.76 (−7.10, −4.41)	−10.69, −0.83	0.472
Santos et al. [41]	2012	LCD (≤26% daily carbohydrate intake)	BW, 6–11 months	MD	−7.44 (−9.07, −5.81)	−13.42, −1.46	0.073
Santos et al. [41]	2012	LCD (≤26% daily carbohydrate intake)	BW, 12–23 months	MD	−6.45 (−8.73, −4.16)	−14.58, 1.69	0.999
Santos et al. [41]	2012	LCD (≤26% daily carbohydrate intake)	BMI, 6 months	MD	−1.72 (−2.28, −1.15)	−8.88, 5.45	0.039
Santos et al. [41]	2012	LCD (≤26% daily carbohydrate intake)	BMI, 6–11 months	MD	−2.03 (−2.62, −1.45)	−4.67, 0.61	0.822
Santos et al. [41]	2012	LCD (≤26% daily carbohydrate intake)	Waist circumference, 6 months	MD	−4.94 (−6.82, −3.05)	−27.75, 17.88	0.129
Santos et al. [41]	2012	LCD (≤26% daily carbohydrate intake)	Waist circumference, 6–11 months	MD	−6.58 (−8.14, −5.02)	−12.35, −0.80	0.628
Santos et al. [41]	2012	LCD (≤26% daily carbohydrate intake)	SBP, 6–11 months	MD	−5.54 (−7.50, −3.57)	−11.88, 0.81	0.594
Santos et al. [41]	2012	LCD (≤26% daily carbohydrate intake)	DBP, 6 months	MD	−3.96 (−5.31, −2.60)	−8.14, 0.22	0.425
Santos et al. [41]	2012	LCD (≤26% daily carbohydrate intake)	DBP, 6–11 months	MD	−3.56 (−4.78, −2.34)	−7.50, 0.38	0.380
Santos et al. [41]	2012	LCD (≤26% daily carbohydrate intake)	HDL-C, 24 months	MD	6.71 (4.80, 8.61)	NA	NA
Santos et al. [41]	2012	LCD (≤26% daily carbohydrate intake)	TG, 6 months	MD	−38.85 (−48.27, −29.43)	−74.41, −3.28	0.756
Santos et al. [41]	2012	LCD (≤26% daily carbohydrate intake)	TG, 6–11 months	MD	−27.61 (−37.38, −17.83)	−60.19, 4.98	0.613
Lari et al. [1]	2021	DASH diet	SBP	MD	−3.94 (−5.24, −2.64)	−9.41, 1.53	0.106
Chiavaroli et al. [22]	2018	Portfolio dietary pattern	LDL-C	SMD	−13.05 (−16.04, −10.06)	−22.01, −4.09	0.006
Chiavaroli et al. [22]	2018	Portfolio dietary pattern	Non-HDL-C	SMD	−14.99 (−18.43, −11.55)	−24.92, −5.06	0.018
Chiavaroli et al. [22]	2018	Portfolio dietary pattern	ApoB	SMD	−18.13 (−22.74, −13.51)	−30.99, −5.27	0.091
Massara et al. [24]	2022	Nordic diet	Stroke incidence (extreme quintiles)	MD	0.87 (0.78, 0.96)	0.68, 1.07	0.148
Massara et al. [24]	2022	Nordic diet	T2D (extreme quintiles)	MD	0.95 (0.85, 1.05)	0.67, 1.23	0.221
Massara et al. [24]	2022	Nordic diet	CVD mortality (extreme quintiles)	MD	0.80 (0.70, 0.90)	0.56, 1.04	0.815
Wang et al. [3]	2015	Vegetarian vs. omnivorous diet	HDL-C	WMD	−1.84 (−2.41, −1.28)	−2.52, −1.16	0.999

Apo B: Apolipoprotein B, BMI: body mass index, BW: body weight, CVD: cardiovascular disease, DBP: diastolic blood pressure, HbA1c: glycated hemoglobin A1c, FPG: fasting plasma glucose, HDL-C: high-density lipoprotein cholesterol, HP: high protein, LCD: low-carbohydrate diet, LDL-C: low-density lipoprotein cholesterol, LGI: low-glycemic index, MD: mean difference, NA: not applicable, SBP: systolic blood pressure, SMD: standardized mean difference, TC: total cholesterol, TG: triglycerides, T2D: type 2 diabetes, WC: waist circumference, WMD: weighted mean difference.

## Data Availability

The data presented in this study are available on request from the corresponding author.

## Supplementary Materials

The following supporting information can be downloaded at: https://www.mdpi.com/article/10.3390/nu16223873/s1, Figure S1. Search Query, Figure S2. Classification Methods Criteria of the Umbrella Review Evidence, Table S1. Results of the umbrella review and Table S2. AMSTAR 2 tool for the Quality assessment of included meta-analyses.

## References

[1] Lari A., Sohouli M.H., Fatahi S., Cerqueira H.S., Santos H.O., Pourrajab B., Rezaei M., Saneie S., Rahideh S.T. (2021). The effects of the Dietary Approaches to Stop Hypertension (DASH) diet on metabolic risk factors in patients with chronic disease: A systematic review and meta-analysis of randomized controlled trials. Nutr. Metab. Cardiovasc. Dis. NMCD.

[2] Dong T., Guo M., Zhang P., Sun G., Chen B. (2020). The effects of low-carbohydrate diets on cardiovascular risk factors: A meta-analysis. PLoS ONE.

[3] Wang F., Zheng J., Yang B., Jiang J., Fu Y., Li D. (2015). Effects of Vegetarian Diets on Blood Lipids: A Systematic Review and Meta-Analysis of Randomized Controlled Trials. J. Am. Heart Assoc..

[4] Kastorini C.M., Milionis H.J., Esposito K., Giugliano D., Goudevenos J.A., Panagiotakos D.B. (2011). The effect of Mediterranean diet on metabolic syndrome and its components: A meta-analysis of 50 studies and 534,906 individuals. J. Am. Coll. Cardiol..

[5] Ojo O., Ojo O.O., Wang X.H., Adegboye A.R.A. (2019). The Effects of a Low GI Diet on Cardiometabolic and Inflammatory Parameters in Patients with Type 2 and Gestational Diabetes: A Systematic Review and Meta-Analysis of Randomised Controlled Trials. Nutrients.

[6] Zhao W.T., Luo Y., Zhang Y., Zhou Y., Zhao T.T. (2018). High protein diet is of benefit for patients with type 2 diabetes: An updated meta-analysis. Medicine.

[7] Huo R., Du T., Xu Y., Xu W., Chen X., Sun K., Yu X. (2015). Effects of Mediterranean-style diet on glycemic control, weight loss and cardiovascular risk factors among type 2 diabetes individuals: A meta-analysis. Eur. J. Clin. Nutr..

[8] Mantzouranis E., Kakargia E., Kakargias F., Lazaros G., Tsioufis K. (2023). The Impact of High Protein Diets on Cardiovascular Outcomes: A Systematic Review and Meta-Analysis of Prospective Cohort Studies. Nutrients.

[9] Fusar-Poli P., Radua J. (2018). Ten simple rules for conducting umbrella reviews. Evid. Based Ment. Health.

[10] Belbasis L., Mavrogiannis M.C., Emfietzoglou M., Evangelou E. (2020). Environmental factors, serum biomarkers and risk of atrial fibrillation: An exposure-wide umbrella review of meta-analyses. Eur. J. Epidemiol..

[11] Aljuraiban G.S., Gibson R., Chan D.S., Van Horn L., Chan Q. (2024). The Role of Diet in the Prevention of Hypertension and Management of Blood Pressure: An Umbrella Review of Meta-Analyses of Interventional and Observational Studies. Adv. Nutr..

[12] Churuangsuk C., Hall J., Reynolds A., Griffin S.J., Combet E., Lean M.E.J. (2022). Diets for weight management in adults with type 2 diabetes: An umbrella review of published meta-analyses and systematic review of trials of diets for diabetes remission. Diabetologia.

[13] Patikorn C., Roubal K., Veettil S.K., Chandran V., Pham T., Lee Y.Y., Giovannucci E.L., Varady K.A., Chaiyakunapruk N. (2021). Intermittent Fasting and Obesity-Related Health Outcomes: An Umbrella Review of Meta-analyses of Randomized Clinical Trials. JAMA Netw. Open.

[14] Patikorn C., Saidoung P., Pham T., Phisalprapa P., Lee Y.Y., Varady K.A., Veettil S.K., Chaiyakunapruk N. (2023). Effects of ketogenic diet on health outcomes: An umbrella review of meta-analyses of randomized clinical trials. BMC Med..

[15] Ocagli H., Berti G., Rango D., Norbiato F., Chiaruttini M.V., Lorenzoni G., Gregori D. (2023). Association of Vegetarian and Vegan Diets with Cardiovascular Health: An Umbrella Review of Meta-Analysis of Observational Studies and Randomized Trials. Nutrients.

[16] Hareer L.W., Lau Y.Y., Mole F., Reidlinger D.P., O’Neill H.M., Mayr H.L., Greenwood H., Albarqouni L. (2024). The effectiveness of the Mediterranean Diet for primary and secondary prevention of cardiovascular disease: An umbrella review. Nutr. Diet. J. Dietit. Assoc. Aust..

[17] Gates M., Gates A., Pieper D., Fernandes R.M., Tricco A.C., Moher D., Brennan S.E., Li T., Pollock M., Lunny C. (2022). Reporting guideline for overviews of reviews of healthcare interventions: Development of the PRIOR statement. BMJ.

[18] Shea B.J., Reeves B.C., Wells G., Thuku M., Hamel C., Moran J., Moher D., Tugwell P., Welch V., Kristjansson E. (2017). AMSTAR 2: A critical appraisal tool for systematic reviews that include randomised or non-randomised studies of healthcare interventions, or both. BMJ.

[19] Papatheodorou S.I., Evangelou E. (2022). Umbrella Reviews: What They Are and Why We Need Them. Methods Mol. Biol..

[20] Higgins J.P.T., Thompson S.G., Deeks J.J., Altman D.G. (2003). Measuring inconsistency in meta-analyses. BMJ.

[21] Markozannes G., Aretouli E., Rintou E., Dragioti E., Damigos D., Ntzani E., Evangelou E., Tsilidis K.K. (2017). An umbrella review of the literature on the effectiveness of psychological interventions for pain reduction. BMC Psychol..

[22] Chiavaroli L., Nishi S.K., Khan T.A., Braunstein C.R., Glenn A.J., Mejia S.B., Rahelić D., Kahleová H., Salas-Salvadó J., Jenkins D.J. (2018). Portfolio Dietary Pattern and Cardiovascular Disease: A Systematic Review and Meta-analysis of Controlled Trials. Prog. Cardiovasc. Dis..

[23] Ajala O., English P., Pinkney J. (2013). Systematic review and meta-analysis of different dietary approaches to the management of type 2 diabetes. Am. J. Clin. Nutr..

[24] Massara P., Zurbau A., Glenn A.J., Chiavaroli L., Khan T.A., Viguiliouk E., Mejia S.B., Comelli E.M., Chen V., Schwab U. (2022). Nordic dietary patterns and cardiometabolic outcomes: A systematic review and meta-analysis of prospective cohort studies and randomised controlled trials. Diabetologia.

[25] Amini M.R., Askarpour M., Ghalandari H., Gholizadeh M., Pouraram H. (2024). Effect of ketogenic diet on blood pressure: A GRADE-Assessed systematic review and meta-analysis of randomized controlled trials. Nutr. Metab. Cardiovasc. Dis..

[26] Choy K.Y.C., Louie J.C.Y. (2023). The effects of the ketogenic diet for the management of type 2 diabetes mellitus: A systematic review and meta-analysis of recent studies. Diabetes Metab. Syndr..

[27] Luo W., Zhang J., Xu D., Zhou Y., Qu Z., Yang Q., Lv Q. (2022). Low carbohydrate ketogenic diets reduce cardiovascular risk factor levels in obese or overweight patients with T2DM: A meta-analysis of randomized controlled trials. Front. Nutr..

[28] Rafiullah M., Musambil M., David S.K. (2022). Effect of a very low-carbohydrate ketogenic diet vs recommended diets in patients with type 2 diabetes: A meta-analysis. Nutr. Rev..

[29] Termannsen A., Clemmensen K.K.B., Thomsen J.M., Nørgaard O., Díaz L.J., Torekov S.S., Quist J.S., Færch K. (2022). Effects of vegan diets on cardiometabolic health: A systematic review and meta-analysis of randomized controlled trials. Obes. Rev..

[30] Chiavaroli L., Lee D., Ahmed A., Cheung A., Khan T.A., Blanco S., Mirrahimi A., Jenkins D.J., Livesey G., Wolever T.M. (2021). Effect of low glycaemic index or load dietary patterns on glycaemic control and cardiometabolic risk factors in diabetes: Systematic review and meta-analysis of randomised controlled trials. BMJ.

[31] López-Espinoza M.Á., Chacón-Moscoso S., Sanduvete-Chaves S., Ortega-Maureira M.J., Barrientos-Bravo T. (2021). Effect of a Ketogenic Diet on the Nutritional Parameters of Obese Patients: A Systematic Review and Meta-Analysis. Nutrients.

[32] Smith E.S., Smith H.A., Betts J.A., Gonzalez J.T., Atkinson G. (2020). A Systematic Review and Meta-Analysis Comparing Heterogeneity in Body Mass Responses Between Low-Carbohydrate and Low-Fat Diets. Obes. Silver.

[33] Ramezani-Jolfaie N., Mohammadi M., Salehi-Abargouei A. (2019). The effect of healthy Nordic diet on cardio-metabolic markers: A systematic review and meta-analysis of randomized controlled clinical trials. Eur. J. Nutr..

[34] Sainsbury E., Kizirian N.V., Partridge S.R., Gill T., Colagiuri S., Gibson A.A. (2018). Effect of dietary carbohydrate restriction on glycemic control in adults with diabetes: A systematic review and meta-analysis. Diabetes Res. Clin. Pract..

[35] Meng Y., Bai H., Wang S., Li Z., Wang Q., Chen L. (2017). Efficacy of low carbohydrate diet for type 2 diabetes mellitus management: A systematic review and meta-analysis of randomized controlled trials. Diabetes Res. Clin. Pract..

[36] Ndanuko R.N., Tapsell L.C., Charlton K.E., Neale E.P., Batterham M.J. (2016). Dietary Patterns and Blood Pressure in Adults: A Systematic Review and Meta-Analysis of Randomized Controlled Trials. Adv. Nutr..

[37] Nissensohn M., Román-Viñas B., Sánchez-Villegas A., Piscopo S., Serra-Majem L. (2016). The Effect of the Mediterranean Diet on Hypertension: A Systematic Review and Meta-Analysis. J. Nutr. Educ. Behav..

[38] Bueno N.B., de Melo I.S.V., de Oliveira S.L., da Rocha Ataide T. (2013). Very-low-carbohydrate ketogenic diet v. low-fat diet for long-term weight loss: A meta-analysis of randomised controlled trials. Br. J. Nutr..

[39] Schwingshackl L., Hoffmann G. (2013). Long-term effects of low-fat diets either low or high in protein on cardiovascular and metabolic risk factors: A systematic review and meta-analysis. Nutr. J..

[40] Fleming P., Godwin M. (2013). Low-glycaemic index diets in the management of blood lipids: A systematic review and meta-analysis. Fam. Pract..

[41] Santos F.L., Esteves S.S., da Costa Pereira A., Yancy W.S., Nunes J.P.L. (2012). Systematic review and meta-analysis of clinical trials of the effects of low carbohydrate diets on cardiovascular risk factors. Obes. Rev. Off. J. Int. Assoc. Study Obes..

[42] Minari T.P., Tácito L.H.B., Yugar L.B.T., Ferreira-Melo S.E., Manzano C.F., Pires A.C., Moreno H., Vilela-Martin J.F., Cosenso-Martin L.N., Yugar-Toledo J.C. (2023). Nutritional Strategies for the Management of Type 2 Diabetes Mellitus: A Narrative Review. Nutrients.

[43] Romero-Cabrera J.L., García-Ríos A., Sotos-Prieto M., Quintana-Navarro G., Alcalá-Díaz J.F., Martín-Piedra L., Torres-Peña J.D., Luque R.M., Yubero-Serrano E.M., Delgado-Lista J. (2023). Adherence to a Mediterranean lifestyle improves metabolic status in coronary heart disease patients: A prospective analysis from the CORDIOPREV study. J. Intern. Med..

[44] Pérez-Martínez P., Mikhailidis D.P., Athyros V.G., Bullo M., Couture P., Covas M.I., de Koning L., Delgado-Lista J., Diaz-Lopez A., Drevon C.A. (2017). Lifestyle recommendations for the prevention and management of metabolic syndrome: An international panel recommendation. Nutr. Rev..

[45] Katsiki N., Stoian A.P., Rizzo M. (2022). Dietary patterns in non-alcoholic fatty liver disease (NAFLD): Stay on the straight and narrow path!. Clin. Investig. Arterioscler. Publ. Soc. Esp. Arterioscler..

[46] Gomez-Delgado F., Katsiki N., Lopez-Miranda J., Perez-Martinez P. (2021). Dietary habits, lipoprotein metabolism and cardiovascular disease: From individual foods to dietary patterns. Crit. Rev. Food Sci. Nutr..

[47] Katsiki N., Td F., Vlachopoulos C., Panagiotakos D., Milionis H., Tselepis A., Garoufi A., Rallidis L., Richter D., Nomikos T. (2024). Executive summary of the Hellenic Atherosclerosis Society guidelines for the diagnosis and treatment of dyslipidemias—2023. Atheroscler. Plus.

[48] Muscogiuri G., Verde L., Sulu C., Katsiki N., Hassapidou M., Frias-Toral E., Cucalón G., Pazderska A., Yumuk V.D., Colao A. (2022). Mediterranean Diet and Obesity-related Disorders: What is the Evidence?. Curr. Obes. Rep..

[49] Ashtary-Larky D., Bagheri R., Bavi H., Baker J.S., Moro T., Mancin L., Paoli A. (2022). Ketogenic diets, physical activity and body composition: A review. Br. J. Nutr..

[50] Dyńka D., Kowalcze K., Charuta A., Paziewska A. (2023). The Ketogenic Diet and Cardiovascular Diseases. Nutrients.

[51] Mateo-Gallego R., Marco-Benedí V., Perez-Calahorra S., Bea A.M., Baila-Rueda L., Lamiquiz-Moneo I., de Castro-Orós I., Cenarro A., Civeira F. (2017). Energy-restricted, high-protein diets more effectively impact cardiometabolic profile in overweight and obese women than lower-protein diets. Clin. Nutr..

[52] Visseren F.L.J., Mach F., Smulders Y.M., Carballo D., Koskinas K.C., Bäck M., Benetos A., Biffi A., Boavida J.-M., Capodanno D. (2021). 2021 ESC Guidelines on cardiovascular disease prevention in clinical practice. Eur. Heart J..

[53] Filippou C., Tatakis F., Polyzos D., Manta E., Thomopoulos C., Nihoyannopoulos P., Tousoulis D., Tsioufis K. (2022). Overview of salt restriction in the Dietary Approaches to Stop Hypertension (DASH) and the Mediterranean diet for blood pressure reduction. Rev. Cardiovasc. Med..

[54] Brown A.G.M., Adas S., de Jesus J., Farmer N., Fisher R., Pratt C.A. (2024). Bridging the Gap: The Need to Implement Dietary Guidance to Address Cardiovascular Health. Nutrients.

[55] EFSA Panel on Dietetic Products, Nutrition, and Allergies (NDA) (2010). Scientific Opinion on establishing Food-Based Dietary Guidelines. EFSA J..

[56] Kahleova H., Salas-Salvadó J., Rahelić D., Kendall C.W., Rembert E., Sievenpiper J.L. (2019). Dietary Patterns and Cardiometabolic Outcomes in Diabetes: A Summary of Systematic Reviews and Meta-Analyses. Nutrients.

[57] Viroli G., Kalmpourtzidou A., Cena H. (2023). Exploring Benefits and Barriers of Plant-Based Diets: Health, Environmental Impact, Food Accessibility and Acceptability. Nutrients.

