# Supplementary material for: Effect of Different Dietary Patterns on Cardiometabolic Risk Factors: An Umbrella Review of Systematic Reviews and Meta-Analyses
Source: Nutrients. 2024 Nov 13;16(22):3873. doi: 10.3390/nu16223873 (PMC11597261; doi:10.3390/nu16223873)
Supplement: Supplementary file 1 [file nutrients-16-03873-s001.zip › nutrients-3221939-supplementary.pdf]

nutrients MDPI

Search Query

((cardiovascular risk) OR hypercholesterolemia OR hyperlipidemia) AND (Diet) AND (systematic review OR meta-analysis)

Figure S1. Search Query.

♦Convincing/ Strong (Class I) when sample size 500, $p<10^{-6}$, $I^2<50\%$, 95% prediction interval excluding the null, no small-study effects and no excess significance bias.
♦Highly suggestive (class II) when sample size >500, $p<10^{-6}$, largest study with a statistically significant effect and class I criteria not met.
♦Suggestive (class III) when sample size >500, $p<10^{-3}$ and class I–II criteria not met.
♦Weak (class IV) when $p<0.05$ and class I–III criteria not met
♦Non-significant when $p>0.05$.

Figure S2. Classification Methods Criteria of the Umbrella Review Evidence.

Table S1. Results of the umbrella review.

Citation	Year	Intervention	Outcome	Units of Measurement	Number of Studies	Total Sample Size	Meta-analysis Metric	Random Effects	P- Value of Random Effects	95% Prediction Interval	I^2	Egger's Test	Grade
Mediterranean Diet													
Huo et al.	2014	Mediterranean Diet	HbA1c	%	11	1359	MD	-0.30 (-0.46, -0.14)	≤0,001	-0.76, 0.16	67	0,001	Suggestive
Huo et al.	2014	Mediterranean Diet	BMI	kg/m2	7	1165	MD	-0.29 (-0.45, -0.13)	0,001	-0.50, -0.08	0	0,481	Suggestive
Huo et al.	2014	Mediterranean Diet	FPI	pmol/L	6	726	MD	-9.91 (-14.53, -5.29)	≤0,001	-16.45, -3.37	0	0,326	Suggestive
Ndanuko et al.	2016	Mediterranean Diet	SBP	mmHg	3	1957	MD	-3.02 (-3.47, -2.58)	≤0,001	-5.89, -0.15	0	0,907	Weak
Ndanuko et al.	2016	Mediterranean Diet	DBP	mmHg	3	1957	MD	-1.99 (-2.27, -1.71)	≤0,001	-3.82, -0.17	0	0,044	Weak
Nissensohn et al.	2016	Mediterranean Diet	SBP	mmHg	7	1957	MD	-3.10 (-6.24, 0.05)	0,05	-14.28, 8.09	98	0,369	Weak
Nissensohn et al.	2016	Mediterranean Diet	DBP	mmHg	7	1957	MD	-0.70 (-1.33, -0.06)	0,03	-2.40, 1.00	63	0,017	Weak
Huo et al.	2014	Mediterranean Diet	TG	mg/dL	7	980	MD	-5.43 (-8.88, -1.99)	≤0,001	-14.62, 3.76	57	0,611	Weak
Huo et al.	2014	Mediterranean Diet	HDL-c	mg/dL	7	980	MD	1.11 (0.40, 1.82)	0,002	-0.76, 2.98	52	0,576	Weak

Huo et al.	2014	Mediterranean Diet	FPG	mg/dL	7	771	MD	-13.04 (-22.37, -3.71)	0,006	-39.72, 13.65	66	0,563	Weak
Huo et al.	2014	Mediterranean Diet	BW	kg	7	980	MD	-0.29 (-0.55, -0.04)	0,022	-0.62, 0.04	0	0,714	Weak
Huo et al.	2014	Mediterranean Diet	TC	mg/dL	7	980	MD	-2.51 (-3.35, -1.66)	0,002	-3.62, -1.39	0	0,031	Weak
Huo et al.	2014	Mediterranean Diet	LDL-c	mg/dL	6	765	MD	-1.95 (-4.19, 0.28)	0,087	-5.13, 1.22	0	0,381	NS
Ajala et al.	2013	Mediterranean Diet	HbA1c	%	3	578	MD	-0.28 (-0.71, 0.15)	0,209	-5.46, 4.90	82	0,164	NS
LCD													
Meng et al.	2017	LCD (≤26% daily carbohydrate intake)	TG	mg/dL	9	724	WMD	-5.81 (-7.96, -3.66)	≤0,001	-8.40, -3.21	0	0,540	Strong
Santos et al.	2012	LCD (≤26% daily carbohydrate intake)	BW , 24 months	kg	4	1572	MD	-4.79 (-5.85, -3.72)	≤0,001	-8.57, -1.00	43	0,709	Strong
Santos et al.	2012	LCD (≤26% daily carbohydrate intake)	SBP, 6 months	mmHg	5	1181	MD	-6.38 (-7.84, -4.93)	≤0,001	-10.04, -2.73	29	0,018	Strong
Santos et al.	2012	LCD (≤26% daily carbohydrate intake)	FPI , 6-11 months	pmol/L	4	1414	MD	-15.35 (-19.58, -11.12)	≤0,001	-24.64, -6.06	0	0,597	Strong
Santos et al.	2012	LCD (≤26% daily carbohydrate intake)	BW , 6-11 months	kg	9	2186	MD	-7.44 (-9.07, -5.81)	≤0,001	-13.42, -1.46	96	0,472	Highly Suggestive

Santos et al.	2012	LCD (≤26% daily carbohydrate intake)	BW , 6 months	kg	8	1081	MD	-5.76 (-7.10, -4.41)	≤0,001	-10.69, -0.83	97	0,073	Highly Suggestive
Santos et al.	2012	LCD (≤26% daily carbohydrate intake)	WC , 6-11 months	cm	5	1374	MD	-6.58 (-8.14, -5.02)	≤0,001	-12.35, -0.80	86	0,628	Highly Suggestive
Santos et al.	2012	LCD (≤26% daily carbohydrate intake)	HDL, 24 months	mg/dL	2	1118	MD	6.71 (4.80, 8.61)	≤0,001	NA	1	NA	Highly Suggestive
Santos et al.	2012	LCD (≤26% daily carbohydrate intake)	DBP, 6 months	mmHg	5	1181	MD	-3.96 (-5.31, -2.60)	≤0,001	-8.14, 0.22	58	0,425	Highly Suggestive
Santos et al.	2012	LCD (≤26% daily carbohydrate intake)	DBP, 6-11 months	mmHg	7	2192	MD	-3.56 (-4.78, -2.34)	≤0,001	-7.50, 0.38	75	0,380	Highly Suggestive
Santos et al.	2012	LCD (≤26% daily carbohydrate intake)	TG, 6-11 months	mg/dL	7	2031	MD	-27.61 (-37.38, -17.83)	≤0,001	-60.19, 4.98	85	0,613	Highly Suggestive
Santos et al.	2012	LCD (≤26% daily carbohydrate intake)	BW , 12-23 months	kg	7	2041	MD	-6.45 (-8.73, -4.16)	≤0,001	-14.58, 1.69	94	0,999	Highly Suggestive
Santos et al.	2012	LCD (≤26% daily carbohydrate intake)	SBP, 6-11 months	mmHg	8	2312	MD	-5.54 (-7.50, -3.57)	≤0,001	-11.88, 0.81	77	0,594	Highly Suggestive
Santos et al.	2012	LCD (≤26% daily carbohydrate intake)	TG, 6 months	mg/dL	4	888	MD	-38.85 (-48.27, -29.43)	≤0,001	-74.41, -3.28	50	0,756	Highly Suggestive

Santos et al.	2012	LCD (≤26% daily carbohydrate intake)	BMI, 6-11 months	kg/m2	4	781	MD	-2.03 (-2.62, -1.45)	≤0,001	-4.67, 0.61	82	0,822	Highly Suggestive
Santos et al.	2012	LCD (≤26% daily carbohydrate intake)	BMI, 6 months	kg/m2	3	581	MD	-1.72 (-2.28, -1.15)	≤0,001	-8.88, 5.45	96	0,039	Highly Suggestive
Santos et al.	2012	LCD (≤26% daily carbohydrate intake)	WC , 6 months	cm	3	563	MD	-4.94 (-6.82, -3.05)	≤0,001	-27.75, 17.88	83	0,129	Highly Suggestive
Meng et al.	2017	LCD (≤26% daily carbohydrate intake)	HbA1c	%	9	724	WMD	-0.43 (-0.63, -0.24)	≤0,001	-0.83, -0.04	20	0,989	Suggestive
Meng et al.	2017	LCD (≤26% daily carbohydrate intake)	HDL	mg/dL	8	622	WMD	1.76 (0.85, 2.66)	≤0,001	-0.12, 3.63	23	0,331	Suggestive
Santos et al.	2012	LCD (≤26% daily carbohydrate intake)	WC , 24 months	2	1133	MD	MD	-4.85 (-6.96, -2.74)	≤0,001	NA	1	NA	Suggestive
Santos et al.	2012	LCD (≤26% daily carbohydrate intake)	WC , 12-23 months	4	1291	MD	MD	-6.79 (-9.94, -3.64)	≤0,001	-21.85, 8.26	96	0,244	Suggestive
Santos et al.	2012	LCD (≤26% daily carbohydrate intake)	SBP, 12-23 months	7	2041	MD	MD	-4.12 (-6.11, -2.13)	≤0,001	-10.44, 2.19	74	0,558	Suggestive
Santos et al.	2012	LCD (≤26% daily carbohydrate intake)	DBP, 12-23 months	8	2161	MD	MD	-2.93 (-4.35, -1.50)	≤0,001	-7.58, 1.73	79	0,785	Suggestive

Santos et al.	2012	LCD (≤26% daily carbohydrate intake)	TG, 12-23 months	6	1230	MD	MD	-23.19 (-35.54, -10.84)	≤0,001	-61.77, 15.40	77	0,742	Suggestive
Santos et al.	2012	LCD (≤26% daily carbohydrate intake)	FPG , 6-11 months	7	2027	MD	MD	-4.00 (-6.38, -1.62)	≤0,001	-11.35, 3.35	79	0,012	Suggestive
Santos et al.	2012	LCD (≤26% daily carbohydrate intake)	FPG , 24 months	mg/dL	2	943	MD	3.50 (1.84, 5.17)	≤0,001	NA	0	NA	Suggestive
Santos et al.	2012	LCD (≤26% daily carbohydrate intake)	BMI, 12-23 months	kg/m2	3	671	MD	-1.41 (-2.15, -0.66)	≤0,001	-10.44, 7.63	83	0,183	Suggestive
Rafiullah et al.	2022	LCD	BW- 3 months	kg	6	797	WMD	-2.91 (-4.88, -0.95)	0,004	-8.44, 2.61	62	0,227	Weak
Rafiullah et al.	2022	LCD	LDL-c - 12 months	mg/dl	4	797	WMD	6.35 (2.02, 10.69)	0,004	-3.16, 15.87	0	0,226	Weak
Rafiullah et al.	2022	LCD	TG- 3 months	mg/dl	4	797	WMD	-34.00 (-58.95, -9.05)	0,008	-88.77, 20.77	0	NA	Weak
Rafiullah et al.	2022	LCD	BW- 6 months	kg	6	797	WMD	-2.84 (-5.29, -0.39)	0,023	-10.91, 5.24	79	0,057	Weak
Rafiullah et al.	2022	LCD	TG- 12 months	mg/dl	5	797	WMD	-24.10 (-33.93, -14.27)	0,000	-40.06, -8.14	0	0,632	Weak
Rafiullah et al.	2022	LCD	HbA1c- 6 months	%	7	797	WMD	-0.58 (-0.84, -0.32)	0,000	-1.31, 0.14	56	0,570	Weak

Rafiullah et al.	2022	LCD	HbA1c- 3 months	%	6	797	WMD	-0.61 (-0.82, -0.40)	0,000	-1.16, -0.06	44	0,892	Weak
Sainsburry et al	2018	LCD	HbA1c- LCD 6 months	%	5	2405	MD	-0.37 (-0.66, -0.08)	0,012	-1.14, 0.39	10	0,035	Weak
Sainsbury et al.	2018	LCD	HbA1c- LCD 3 months	%	4	2405	MD	-0.47 (-0.71, -0.23)	≤0,000	-0.99, 0.05	0	0,583	Weak
Ndanuko et al.	2016	LCD	HbA1c- low carbo 6 months	%	5	1957	MD	-0.36 (-0.62, -0.09)	0,008	-0.79, 0.07	0	0,207	Weak
Ndanuko et al.	2016	LCD	HbA1c- moderate carbo 6months	%	6	1957	MD	-0.06 (-0.25, 0.13)	0,550	-0.61, 0.49	60	0,959	Weak
Ndanuko et al.	2016	LCD	HbA1c- low carbo 3 months	%	4	1957	MD	-0.47 (-0.71, -0.23)	1,1E-04	-0.99, 0.05	0	0,583	Weak
Bueno et al.	2013	LCD	BW	kg	13	1577	MD	-1.17 (-1.92, -0.43)	0,002	-2.01, -0.34	0	0,593	Weak
Bueno et al.	2013	LCD	LDL-c	mg/Dl	12	1577	MD	2.16 (0.77, 3.54)	0,002	0.58, 3.73	0	0,453	Weak
Bueno et al.	2013	LCD	TAG	mg/Dl	12	1577	MD	-3.15 (-4.85, -1.45)	≤0,000	-6.23, -0.08	13	0,043	Weak
Bueno et al.	2013	LCD	HDL-c	mg/Dl	12	1577	MD	1.66 (1.16, 2.16)	≤0,000	0.90, 2.42	6	0,549	Weak
Santos et al.	2012	LCD (≤26% daily carbohydrate intake)	FPI , 6 months	pmol/L	2	360	MD	-18.55 (-23.59, -13.50)	≤0,001	NA	0	NA	Weak

Santos et al.	2012	LCD (≤26% daily carbohydrate intake)	HbA1c, 12-23 months	%	3	452	MD	-0.29 (-0.48, -0.11)	0,002	-2.32, 1.73	60	0,209	Weak
Santos et al.	2012	LCD (≤26% daily carbohydrate intake)	FPI , 12-23 months	pmol/L	3	480	MD	-10.45 (-17.48, -3.42)	0,004	-80.65, 59.75	49	0,485	Weak
Santos et al.	2012	LCD (≤26% daily carbohydrate intake)	FPG , 12-23 months	mg/dL	4	791	MD	-3.68 (-6.74, -0.62)	0,018	-14.81, 7.44	48	0,914	Weak
Santos et al.	2012	LCD (≤26% daily carbohydrate intake)	TG, 24 months	mg/dL	2	1118	MD	-21.64 (-39.93, -3.34)	0,020	NA	1	NA	Weak
Santos et al.	2012	LCD (≤26% daily carbohydrate intake)	LDL-c, 24 months	mg/dL	2	1118	MD	-3.27 (-6.16, -0.38)	0,026	NA	0	NA	Weak
Santos et al.	2012	LCD (≤26% daily carbohydrate intake)	HDL-c, 12-23 months	mg/dL	6	1230	MD	3.07 (0.33, 5.81)	0,028	-6.88, 13.02	94	0,481	Weak
Santos et al.	2012	LCD (≤26% daily carbohydrate intake)	SBP, 24 months	mmHg	2	1118	MD	-1.57 (-2.99, -0.14)	0,032	NA	0	NA	Weak
Santos et al.	2012	LCD (≤26% daily carbohydrate intake)	CRP, 12-23 months	mg/dL	2	280	MD	-0.07 (-0.14, -0.00)	0,035	NA	0	NA	Weak
Santos et al.	2012	LCD (≤26% daily carbohydrate intake)	LDL-c, 6 months	mg/dL	7	1046	MD	2.31 (0.06, 4.56)	0,044	-2.12, 6.74	18	0,425	Weak

Santos et al.	2012	LCD (≤26% daily carbohydrate intake)	HDL-c, 6 months	mg/dL	7	1046	MD	1.49 (0.00, 2.98)	0,050	-3.67, 6.66	88	0,031	Weak
Rafiullah et al.	2022	LCD	TG- 6 months	mg/dl	7	797	WMD	-20.48 (-41.17, 0.20)	0,052	-86.40, 45.43	84	0,775	NS
Rafiullah et al.	2022	LCD	LDL-c - 6 months	mg/dl	6	797	WMD	6.34 (-0.88, 13.56)	0,085	-17.27, 29.96	78	0,363	NS
Rafiullah et al.	2022	LCD	HbA1c- 12 months	%	5	797	WMD	-0.17 (-0.47, 0.12)	0,254	-1.04, 0.69	47	0,663	NS
Rafiullah et al.	2022	LCD	LDL-c - 3 months	mg/dl	3	797	WMD	0.62 (-2.38, 3.62)	0,686	-18.85, 20.09	0	0,616	NS
Rafiullah et al.	2022	LCD	BW- 12 months	kg	4	797	WMD	-0.03 (-2.35, 2.29)	0,978	-9.18, 9.12	59	0,427	NS
Sainsbury et al.	2018	LCD	HbA1c- LCD 12 months	%	4	2405	MD	-0.18 (-0.44, 0.08)	0,184	-0.75, 0.40	0	0,422	NS
Sainsbury et al.	2018	LCD	HbA1c- MODERATE 12 months	%	8	2405	MD	-0.09 (-0.23, 0.06)	0,247	-0.41, 0.24	30	0,084	NS
Sainsbury et al.	2018	LCD	HbA1c- MODERATE 3 months	%	8	2405	MD	-0.06 (-0.17, 0.06)	0,337	-0.20, 0.09	0	0,010	NS
Sainsbury et al	2018	LCD	HbA1c- MODERATE 6 months	%	6	2405	MD	-0.06 (-0.25, 0.13)	0,550	-0.61, 0.49	60	0,959	NS
Meng et al.	2017	LCD (≤26% daily carbohydrate intake)	TC	mg/dL	6	564	WMD	1.49 (-0.99, 3.97)	0,240	-2.03, 5.00	0	0,393	NS

Meng et al.	2017	LCD (≤26% daily carbohydrate intake)	BW	kg	8	580	WMD	-0.69 (-2.15, 0.78)	0,360	-4.04, 2.67	35	0,799	NS
Meng et al.	2017	LCD (≤26% daily carbohydrate intake)	FPG	mg/dL	5	432	WMD	-4.26 (-13.64, 5.12)	0,374	-19.49, 10.97	0	0,693	NS
Meng et al.	2017	LCD (≤26% daily carbohydrate intake)	LDL-c	mg/dL	7	570	WMD	0.69 (-1.53, 2.92)	0,541	-2.22, 3.61	0	0,404	NS
Meng et al.	2017	LCD (≤26% daily carbohydrate intake)	BW , <12 months	kg	5	317	WMD	-0.68 (-2.86, 1.50)	0,543	-7.05, 5.70	56	0,685	NS
Meng et al.	2017	LCD (≤26% daily carbohydrate intake)	BW , >- 12 months	kg	3	263	WMD	-0.24 (-2.19, 1.70)	0,806	-12.84, 12.35	0	0,656	NS
Ndanuko et al.	2016	LCD	HbA1c- low carbo 12 months	%	4	NR	MD	-0.18 (-0.44, 0.08)	0,184	-0.75, 0.40	0	0,422	Weak
Ndanuko et al.	2016	LCD	HbA1c- moderate carbo 12 months	%	8	NR	MD	-0.09 (-0.23, 0.06)	0,247	-0.41, 0.24	30	0,084	Weak
Ndanuko et al.	2016	LCD	HbA1c- moderate carbo 3 months	%	8	NR	MD	-0.06 (-0.17, 0.06)	0,337	-0.20, 0.09	0	0,010	Weak
Ajala et al.	2013	LCD	HbA1c	%	8	866	MD	-0.26 (-0.54, 0.02)	0,069	-1.10, 0.58	71	0,164	NS

Bueno et al.	2013	LCD	SBP	mmHg	11	1577	MD	-1.46 (-3.59, 0.68)	0,181	-7.13, 4.22	44	0,869	NS
Bueno et al.	2013	LCD	DBP	mmHg	11	1577	MD	-0.53 (-1.98, 0.92)	0,476	-3.99, 2.94	34	0,056	NS
Santos et al.	2012	LCD (≤26% daily carbohydrate intake)	HDL, 6-11 months	mg/dL	7	2031	MD	2.09 (-0.04, 4.22)	0,054	-5.51, 9.69	93	0,809	NS
Santos et al.	2012	LCD (≤26% daily carbohydrate intake)	HbA1c, 6-11 months	%	2	332	MD	-0.29 (-0.63, 0.04)	0,083	NA	0	NA	NS
Santos et al.	2012	LCD (≤26% daily carbohydrate intake)	DBP, 24 months	mmHg	2	1118	MD	-1.72 (-4.54, 1.10)	0,232	NA	1	NA	NS
Santos et al.	2012	LCD (≤26% daily carbohydrate intake)	LDL-c, 12-23 months	mg/dL	6	1230	MD	-2.08 (-6.36, 2.21)	0,342	-15.49, 11.33	68	0,416	NS
Santos et al.	2012	LCD (≤26% daily carbohydrate intake)	FPG , 6 months	mg/dL	3	764	MD	-0.93 (-2.87, 1.01)	0,348	-20.14, 18.28	48	0,027	NS
Santos et al.	2012	LCD (≤26% daily carbohydrate intake)	FPI , 24 months	pmol/L	2	943	MD	20.98 (-40.51, 82.46)	0,504	NA	1	NA	NS
Santos et al.	2012	LCD (≤26% daily carbohydrate intake)	LDL-c, 6-11 months	mg/dL	7	2031	MD	-0.29 (-1.98, 1.41)	0,741	-2.68, 2.11	2	0,434	NS
Low GI Diet													

Chiavaroli et al.	2021	Low GI Diet	BW (T2D)	-	23	1433	SMD	-0.66 (-0.90, -0.43)	≤0,001	-0.91, -0.41	0	0,709	Strong
Chiavaroli et al.	2021	Low GI Diet	FPG (T2D)	-	22	1455	SMD	-5.86 (-8.10, -3.62)	≤0,001	-12.48, 0.76	53	0,004	Highly Suggestive
Chiavaroli et al.	2021	Low GI Diet	HbA1c (T2D)	-	18	1465	SMD	-0.32 (-0.45, -0.19)	≤0,001	-0.79, 0.15	79	0,208	Suggestive
Chiavaroli et al.	2021	Low GI Diet	LDL-c (T2D)	-	22	1460	SMD	-3.32 (-4.98, -1.66)	≤0,001	-9.06, 2.42	70	0,130	Suggestive
Chiavaroli et al.	2021	Low GI Diet	FPG (T1D)	-	3	84	SMD	-32.59 (-33.93, -31.25)	≤0,001	-41.27, -23.91	0	0,808	Weak
Chiavaroli et al.	2021	Low GI Diet	Non-HDL-c (T2D)	-	21	1420	SMD	-3.95 (-6.37, -1.53)	0,001	-13.51, 5.60	75	0,057	Weak
Chiavaroli et al.	2021	Low GI Diet	BMI (T2D)	-	20	1363	SMD	-0.38 (-0.63, -0.13)	0,003	-0.65, -0.11	0	0,090	Weak
Chiavaroli et al.	2021	Low GI Diet	Apo B (T2D)	-	4	247	SMD	-5.47 (-9.87, -1.08)	0,015	-23.23, 12.28	68	0,850	Weak
Chiavaroli et al.	2021	Low GI Diet	CRP (T2D)	-	6	522	SMD	-0.04 (-0.08, -0.00)	0,03	-0.12, 0.04	24	0,720	Weak
Chiavaroli et al	2021	Low GI Diet	HbA1c (T1D)	-	3	157	SMD	-0.29 (-0.56, -0.02)	0,038	-2.06, 1.48	23	0,381	Weak
Fleming & Godwin	2013	Low GI Diet	LDL-c	mg/dL	4	212	MD	-4.86 (-7.91, -1.81)	0,002	-11.55, 1.83	0	0,266	Weak
Fleming & Godwin	2013	Low GI Diet	HDL-c	mg/dL	4	212	MD	-4.12 (-7.09, -1.16)	0,006	-10.63, 2.38	0	0,917	Weak

Chiavaroli et al	2021	Low GI Diet	TG (T2D)	-	22	1460	SMD	-1.46 (-3.09, 0.17)	0,08	-6.37, 3.46	45	0,505	NS
Chiavaroli et al.	2021	Low GI Diet	WC (T2D)	-	10	918	SMD	-0.67 (-1.76, 0.41)	0,226	-3.69, 2.34	79	0,230	NS
Chiavaroli et al.	2021	Low GI Diet	Non-HDL-c (T1D)	-	3	84	SMD	-3.70 (-10.81, 3.42)	0,308	-76.89, 69.49	51	0,721	NS
Chiavaroli et al. (	2021	Low GI Diet	FPI (T2DM)	-	12	892	SMD	-2.66 (-8.82, 3.49)	0,397	-16.79, 11.46	38	0,024	NS
Chiavaroli et al.	2021	Low GI Diet	LDL-c (T1D)	-	3	84	SMD	-3.20 (-11.43, 5.03)	0,446	-99.25, 92.85	75	0,726	NS
Chiavaroli et al.	2021	Low GI Diet	TG (T1D)	-	3	84	SMD	-1.16 (-4.18, 1.85)	0,450	-32.88, 30.56	54	0,368	NS
Chiavaroli et al	2021	Low GI Diet	DBP (T2D)	-	8	716	SMD	-0.50 (-1.85, 0.86)	0,474	-4.33, 3.34	63	0,235	NS
Chiavaroli et al.	2021	Low GI Diet	HDL-c (T1D)	-	3	84	SMD	-2.23 (-8.38, 3.92)	0,477	-71.66, 67.20	71	0,580	NS
Chiavaroli et al.	2021	Low GI Diet	HDL-c (T2D)	-	22	1460	SMD	0.12 (-0.27, 0.50)	0,554	-0.98, 1.21	53	0,551	NS
Chiavaroli et al.	2021	Low GI Diet	SBP (T2D)	-	9	819	SMD	-0.14 (-2.24, 1.96)	0,894	-5.69, 5.41	53	0,262	NS
Ojo et al.	2019	Low GI Diet	TC (T2D)	mg/dL	6	452	WMD	-1.84 (-6.14, 2.46)	0,402	-7.93, 4.26	0	0,866	NS
Ojo et al	2019	Low GI Diet	LDL (T2D)	mg/dL	5	432	WMD	-2.54 (-6.71, 1.63)	0,233	-9.31, 4.23	0	0,241	NS

Ojo et al.	2019	Low GI Diet	TG (T2D)	mg/dL	6	452	WMD	-0.05 (-2.86, 2.76)	0,972	-6.60, 6.50	27	0,575	NS
Ojo et al.	2019	Low GI Diet	HDL-c (T2D)	mg/dL	7	662	WMD	0.00 (-0.29, 0.29)	1	-0.42, 0.42	0	NA	NS
Ajala et al.	2013	Low GI Diet	HbA1c	%	3	357	MD	-0.12 (-0.39, 0.15)	0,382	-3.20, 2.96	80	0,879	NS
Fleming & Godwin	2013	Low GI Diet	TC	mg/dL	4	212	MD	1.36 (-1.40, 4.12)	0,334	-10.67, 13.39	81	0,981	NS
Fleming & Godwin	2013	Low GI Diet	TG	mg/dL	4	212	MD	-2.73 (-8.70, 3.23)	0,369	-29.56, 24.10	84	0,471	NS
Ketogenic Diet													
Choy & Louie	2023	Ketogenic Diet	TG	-	11	518	SMD	-0.42 (-0.64, -0.19)	≤0,001	-0.95, 0.11	31	0,571	Suggestive
Choy & Louie	2023	Ketogenic Diet	TG (6-12 months)	-	6	312	SMD	-0.41 (-0.64, -0.18)	≤0,000	-0.76, -0.06	2	0,438	Weak
Choy & Louie	2023	Ketogenic Diet	LDL-c (6-12 months)	-	5	282	SMD	0.34 (0.10, 0.57)	0,005	-0.05, 0.72	0	0,446	Weak
Choy & Louie	2023	Ketogenic Diet	FPI (0-3 months)	-	2	83	SMD	-0.57 (-1.01, -0.13)	0,011	NA	0	NA	Weak
Choy & Louie	2023	Ketogenic Diet	BW (3-6 months)	-	8	427	SMD	-0.29 (-0.51, -0.07)	0,011	-0.76, 0.18	23	0,998	Weak
Choy & Louie	2023	Ketogenic Diet	HDL-c (12-24 months)	-	13	700	SMD	0.19 (0.04, 0.34)	0,013	0.02, 0.36	0	0,863	Weak

Choy & Louie	2023	Ketogenic Diet	HbA1c (3-6 months)	-	7	332	SMD	-0.65 (-1.19, -0.10)	0,020	-2.48, 1.19	81	0,982	Weak
Choy & Louie	2023	Ketogenic Diet	FPI (3-6 months)	-	4	189	SMD	-0.32 (-0.61, -0.04)	0,027	-0.96, 0.31	0	0,617	Weak
Choy & Louie	2023	Ketogenic Diet	SBP (3-6 months)	-	6	341	SMD	-0.24 (-0.47, -0.02)	0,031	-0.56, 0.07	0	0,494	Weak
Choy & Louie	2023	Ketogenic Diet	HbA1c	-	11	518	SMD	-0.33 (-0.64, -0.02)	0,038	-1.32, 0.66	64	0,409	Weak
Luo et al.	2022	Ketogenic Diet	HbA1c obese with T2D	%	4	274	WMD	-0.53 (-0.78, -0.29)	≤0,000	-1.07, 0.01	0	0,543	Weak
Luo et al.	2022	Ketogenic Diet	BW obese with T2D	kg	4	245	WMD	-6.77 (-10.20, -3.34)	≤0,000	-14.29, 0.75	0	0,114	Weak
Luo et al.	2022	Ketogenic Diet	HDL-c obese	mg/dL	10	535	WMD	2.28 (0.72, 3.85)	0,004	-2.72, 7.29	81	0,027	Weak
Luo et al.	2022	Ketogenic Diet	FPG obese with T2DM	mg/dL	5	287	WMD	-12.67 (-21.69, -3.65)	0,006	-27.32, 1.98	0	0,631	Weak
Luo et al.	2022	Ketogenic Diet	Body Fat Volume obese	kg	4	102	WMD	-1.48 (-2.55, -0.40)	0,007	-3.84, 0.89	0	0,073	Weak
Luo et al.	2022	Ketogenic Diet	TG obese with T2D	mg/dL	5	277	WMD	-5.73 (-10.26, -1.20)	0,013	-19.07, 7.61	51	0,921	Weak
Luo et al.	2022	Ketogenic Diet	BMI obese with T2D	kg/m2	3	166	WMD	-2.27 (-4.33, -0.20)	0,032	-15.66, 11.13	0	0,860	Weak
Smith et al.	2020	Ketogenic Diet	BW (no ketogenic)	kg	20	3340	MD	-1.21 (-2.01, -0.41)	0,003	-4.23, 1.82	70	0,288	Weak

Amini et al.	2024	Ketogenic Diet	DBP	mmHg	24	1668	MD	-0.11 (-1.15, 0.93)	0,841	-3.45, 3.24	43	0,275	NS
Amini et al.	2024	Ketogenic Diet	SBP	mmHg	23	1652	MD	-0.87 (-2.05, 0.31)	0,148	-3.54, 1.80	17	0,961	NS
Choy & Louie	2023	Ketogenic Diet	Glomerular Filtration (6-12 months)	-	2	159	SMD	-0.30 (-0.61, 0.01)	0,057	NA	0	NA	NS
Choy & Louie	2023	Ketogenic Diet	TC (6-12 months)	-	4	259	SMD	0.55 (-0.14, 1.24)	0,118	-7.47, 8.57	74	0,163	NS
Choy & Louie	2023	Ketogenic Diet	HOMA(3-6 months)	-	3	201	SMD	-0.22 (-0.50, 0.06)	0,119	-2.02, 1.58	0	0,697	NS
Choy & Louie	2023	Ketogenic Diet	FPI	-	4	215	SMD	-0.21 (-0.48, 0.06)	0,120	-0.81, 0.38	0	0,636	NS
Choy & Louie	2023	Ketogenic Diet	Urinary Albumin (3-6 months)	-	1	29	SMD	-0.59 (-1.34, 0.16)	0,123	NA	0	NA	NS
Choy & Louie	2023	Ketogenic Diet	FPG	-	8	450	SMD	-0.35 (-0.81, 0.10)	0,127	-1.88, 1.17	81	0,095	NS
Choy & Louie	2023	Ketogenic Diet	HbA1c (12-24 months)	-	2	183	SMD	0.37 (-0.12, 0.85)	0,135	NA	0	NA	NS
Choy & Louie	2023	Ketogenic Diet	HOMA (0-3 months)	-	1	33	SMD	-0.53 (-1.23, 0.16)	0,135	NA	0	NA	NS
Choy & Louie	2023	Ketogenic Diet	SBP	-	7	377	SMD	-0.44 (-1.01, 0.14)	0,136	-2.41, 1.54	86	0,568	NS
Choy & Louie	2023	Ketogenic Diet	WC (3-6 months)	-	2	135	SMD	-0.47 (-1.10, 0.15)	0,137	NA	1	NA	NS

Choy & Louie	2023	Ketogenic Diet	BW	-	10	495	SMD	-0.22 (-0.51, 0.08)	0,151	-1.12, 0.69	63	0,221	NS
Choy & Louie	2023	Ketogenic Diet	WC	-	3	250	SMD	-0.33 (-0.78, 0.13)	0,156	-5.43, 4.77	67	0,894	NS
Choy & Louie	2023	Ketogenic Diet	TG (12-24 months)	-	2	183	SMD	-0.62 (-1.48, 0.24)	0,158	NA	1	NA	NS
Choy & Louie	2023	Ketogenic Diet	FPG (3-6 months)	-	6	337	SMD	-0.14 (-0.35, 0.07)	0,179	-0.55, 0.26	15	0,140	NS
Choy & Louie	2023	Ketogenic Diet	BMI (0-3 months)	-	3	95	SMD	-0.27 (-0.68, 0.13)	0,188	-2.92, 2.37	0	0,831	NS
Choy & Louie	2023	Ketogenic Diet	BMI	-	7	378	SMD	-0.37 (-0.96, 0.23)	0,229	-2.41, 1.68	86	0,815	NS
Choy & Louie	2023	Ketogenic Diet	BW (0-3 months)	-	4	139	SMD	-0.20 (-0.54, 0.13)	0,235	-0.94, 0.53	0	0,300	NS
Choy & Louie	2023	Ketogenic Diet	Creatinine (6-12 months)	-	2	161	SMD	0.19 (-0.12, 0.50)	0,237	NA	0	NA	NS
Choy & Louie	2023	Ketogenic Diet	BMI (3-6 months)	-	5	298	SMD	-0.45 (-1.22, 0.31)	0,246	-3.35, 2.44	90	0,991	NS
Choy & Louie	2023	Ketogenic Diet	Urinary Albumin (0-3 months)	-	1	35	SMD	0.38 (-0.29, 1.05)	0,266	NA	0	NA	NS
Choy & Louie	2023	Ketogenic Diet	FPG (6-12 months)	-	4	259	SMD	-0.50 (-1.41, 0.40)	0,276	-4.76, 3.75	91	0,201	NS
Choy & Louie	2023	Ketogenic Diet	TC	-	9	465	SMD	0.09 (-0.09, 0.28)	0,314	-0.13, 0.32	0	0,853	NS

Choy & Louie	2023	Ketogenic Diet	SBP (12-24 months)	-	2	200	SMD	-0.92 (-2.90, 1.06)	0,364	NA	1	NA	NS
Choy & Louie	2023	Ketogenic Diet	DBP (12-24 months)	-	2	200	SMD	0.13 (-0.16, 0.43)	0,367	NA	0	NA	NS
Choy & Louie	2023	Ketogenic Diet	HbA1c (0-3 months)	-	4	143	SMD	-0.26 (-0.82, 0.31)	0,370	-2.56, 2.05	63	0,979	NS
Choy & Louie	2023	Ketogenic Diet	DBP (3-6 months)	-	6	341	SMD	0.34 (-0.43, 1.11)	0,387	-2.43, 3.11	91	0,388	NS
Choy & Louie	2023	Ketogenic Diet	Flow-mediated Dilation	-	2	149	SMD	-0.25 (-0.82, 0.32)	0,391	NA	1	NA	NS
Choy & Louie	2023	Ketogenic Diet	TC (12-24 months)	-	2	183	SMD	0.12 (-0.17, 0.41)	0,432	NA	0	NA	NS
Choy & Louie	2023	Ketogenic Diet	FPI (6-12 months)	-	2	140	SMD	-0.13 (-0.46, 0.20)	0,433	NA	0	NA	NS
Choy & Louie	2023	Ketogenic Diet	Urinary Albumin (6-12 months)	-	3	186	SMD	0.17 (-0.26, 0.60)	0,435	-4.16, 4.50	46	0,539	NS
Choy & Louie	2023	Ketogenic Diet	HOMA (6-12 months)	-	2	140	SMD	-0.13 (-0.46, 0.20)	0,450	NA	0	NA	NS
Choy & Louie	2023	Ketogenic Diet	Urinary Albumin	-	3	186	SMD	0.17 (-0.28, 0.61)	0,465	-4.39, 4.72	49	0,529	NS
Choy & Louie	2023	Ketogenic Diet	HOMA (12-24 months)	-	1	108	SMD	-0.14 (-0.52, 0.24)	0,470	NA	0	NA	NS
Choy & Louie	2023	Ketogenic Diet	Glomerular Filtration (12-24 months)	-	1	113	SMD	-0.13 (-0.50, 0.24)	0,485	NA	0	NA	NS

Choy & Louie	2023	Ketogenic Diet	LDL-c (0-3 months)	-	2	45	SMD	0.20 (-0.39, 0.79)	0,499	NA	0	NA	NS
Choy & Louie	2023	Ketogenic Diet	DBP (0-3 months)	-	2	77	SMD	0.15 (-0.29, 0.60)	0,500	NA	0	NA	NS
Choy & Louie	2023	Ketogenic Diet	Glomerular Filtration	-	2	159	SMD	-0.10 (-0.41, 0.20)	0,507	NA	0	NA	NS
Choy & Louie	2023	Ketogenic Diet	HbA1c (6-12 months)	-	6	312	SMD	-0.22 (-0.89, 0.46)	0,525	-2.57, 2.13	87	0,404	NS
Choy & Louie	2023	Ketogenic Diet	TC (0-3 months)	-	2	49	SMD	-0.18 (-0.74, 0.38)	0,529	NA	0	NA	NS
Choy & Louie	2023	Ketogenic Diet	BW (12-24 months)	-	2	183	SMD	-0.09 (-0.38, 0.20)	0,551	NA	0	NA	NS
Choy & Louie	2023	Ketogenic Diet	Flow-mediated Dilation (6-12 months)	-	1	116	SMD	0.11 (-0.26, 0.48)	0,555	NA	0	NA	NS
Choy & Louie	2023	Ketogenic Diet	DBP	-	7	377	SMD	-0.08 (-0.34, 0.18)	0,556	-0.70, 0.55	34	0,410	NS
Choy & Louie	2023	Ketogenic Diet	Urinary Albumin (12-24 months)	-	1	113	SMD	-0.11 (-0.48, 0.26)	0,560	NA	0	NA	NS
Choy & Louie	2023	Ketogenic Diet	FPI (12-24 months)	-	1	108	SMD	-0.11 (-0.49, 0.27)	0,570	NA	0	NA	NS
Choy & Louie	2023	Ketogenic Diet	BW (6-12 months)	-	2	183	SMD	-0.09 (-0.38, 0.20)	0,551	NA	0	0,224	NS

Choy & Louie	2023	Ketogenic Diet	DBP	-	7	377	SMD	-0.08 (-0.34, 0.18)	0,556	-0.70, 0.55	34	0,410	NS
Choy & Louie	2023	Ketogenic Diet	LDL-c (12-24 months)	-	2	183	SMD	-0.12 (-0.57, 0.34)	0,617	NA	1	NA	NS
Choy & Louie	2023	Ketogenic Diet	WC (6-12 months)	--	1	115	SMD	-0.09 (-0.46, 0.28)	0,629	NA	0	NA	NS
Choy & Louie	2023	Ketogenic Diet	CRP (12-24 months)	-	1	112	SMD	0.09 (-0.28, 0.46)	0,634	NA	0	NA	NS
Choy & Louie	2023	Ketogenic Diet	DBP (6-12 months)	-	5	293	SMD	0.20 (-0.65, 1.06)	0,639	-3.04, 3.45	91	0,938	NS
Choy & Louie	2023	Ketogenic Diet	BMI (12-24 months)	-	1	115	SMD	0.08 (-0.29, 0.45)	0,668	NA	0	NA	NS
Choy & Louie	2023	Ketogenic Diet	WC (12-24 months)	-	2	183	SMD	-0.08 (-0.45, 0.29)	0,668	NA	0	NA	NS
Choy & Louie	2023	Ketogenic Diet	CRP (0-3 months)	-	1	33	SMD	-0.15 (-0.83, 0.53)	0,668	NA	0	NA	NS
Choy & Louie	2023	Ketogenic Diet	TC (3-6 months)	-	7	380	SMD	0.04 (-0.16, 0.24)	0,668	-0.22, 0.31	0	0,973	NS
Choy & Louie	2023	Ketogenic Diet	SBP (6-12 months)	-	5	293	SMD	0.18 (-0.63, 0.98)	0,668	-2.86, 3.21	90	0,880	NS
Choy & Louie	2023	Ketogenic Diet	HDL-c (0-3 months)	-	3	79	SMD	0.12 (-0.46, 0.70)	0,688	-5.33, 5.57	36	0,439	NS
Choy & Louie	2023	Ketogenic Diet	BMI (6-12 months)	-	3	195	SMD	0.05 (-0.23, 0.33)	0,726	-1.77, 1.87	0	0,359	NS

Choy & Louie	2023	Ketogenic Diet	CRP (6-12 months)	-	3	195	SMD	0.06 (-0.27, 0.38)	0,738	NA	0	NA	NS
Choy & Louie	2023	Ketogenic Diet	CRP (3-6 motnhs)	-	2	146	SMD	-0.04 (-0.35, 0.27)	0,787	-2.05, 1.96	0	0,288	NS
Choy & Louie	2023	Ketogenic Diet	LDL-c (3-6 months)	-	3	163	SMD	-0.04 (-0.36, 0.28)	0,799	-0.95, 0.87	0	0,493	NS
Choy & Louie	2023	Ketogenic Diet	LDL-c	-	8	410	SMD	0.03 (-0.25, 0.31)	0,808	-0.76, 0.83	57	0,829	NS
Choy & Louie	2023	Ketogenic Diet	SBP (0-3 months)	-	10	488	SMD	-0.05 (-0.50, 0.39)	0,815	NA	53	NA	NS
Choy & Louie	2023	Ketogenic Diet	CRP	-	2	77	SMD	0.03 (-0.26, 0.32)	0,825	-1.85, 1.92	0	0,020	NS
Choy & Louie	2023	Ketogenic Diet	BMI (6-12 months)	-	3	182	SMD	0.05 (-0.23, 0.33)	0,726	-1.77, 1.87	0	0,359	NS
Choy & Louie	2023	Ketogenic Diet	FPG (0-3 months)	-	3	116	SMD	0.02 (-0.34, 0.39)	0,896	-2.36, 2.41	0	0,873	NS
Choy & Louie	2023	Ketogenic Diet	Flow-mediated Dilation (12-24 months)	-	1	113	SMD	-0.02 (-0.39, 0.34)	0,896	NA	0	NA	NS
Choy & Louie	2023	Ketogenic Diet	TG (3-6 months)	-	8	353	SMD	-0.01 (-0.33, 0.31)	0,914	-0.92, 0.90	53	0,474	NS
Choy & Louie	2023	Ketogenic Diet	HDL-c (3-6 months)	-	9	413	SMD	-0.00 (-0.44, 0.43)	0,987	-1.47, 1.46	78	0,994	NS

Choy & Louie	2023	Ketogenic Diet	Creatinine (12-24 months)	-	1	115	SMD	0.00 (-0.37, 0.37)	1	NA	0	NA	NS
Choy & Louie	2023	Ketogenic Diet	Creatinine	-	2	159	SMD	0.00 (-0.31, 0.31)	1	NA	0	NA	NS
Luo et al.	2022	Ketogenic Diet	HDL-c obese with T2D	mg/dL	4	245	WMD	1.24 (-0.00, 2.48)	0,051	-1.49, 3.96	0	0,746	NS
Luo et al.	2022	Ketogenic Diet	BMI obese	kg/m2	6	193	WMD	-0.81 (-1.65, 0.02)	0,056	-1.99, 0.37	0	0,702	NS
Luo et al.	2022	Ketogenic Diet	TG obese	mg/dL	10	454	WMD	-2.74 (-5.59, 0.10)	0,059	-11.96, 6.48	82	0,058	NS
Luo et al.	2022	Ketogenic Diet	TC obese with T2D	mg/dL	3	192	WMD	-2.87 (-6.45, 0.70)	0,115	-26.03, 20.28	0	0,135	NS
Luo et al.	2022	Ketogenic Diet	FPG obese	mg/dL	11	488	WMD	-2.83 (-6.37, 0.70)	0,116	-15.88, 10.21	93	0,346	NS
Luo et al.	2022	Ketogenic Diet	TC obese	mg/dL	10	444	WMD	6.25 (-1.62, 14.12)	0,120	-22.28, 34.78	93	0,011	NS
Luo et al.	2022	Ketogenic Diet	HOMA obese with T2D	.	2	122	WMD	-0.60 (-1.36, 0.16)	0,121	NA	1	NA	NS
Luo et al	2022	Ketogenic Diet	LDL-c obese	mg/dL	10	454	WMD	4.38 (-1.37, 10.12)	0,135	-16.33, 25.08	94	0,045	NS
Luo et al.	2022	Ketogenic Diet	LDL-c obese with T2D	mg/dL	4	245	WMD	-2.38 (-5.78, 1.01)	0,169	-9.84, 5.07	0	0,686	NS
Luo et al.	2022	Ketogenic Diet	SBP obese	mmHg	4	293	WMD	1.56 (-0.69, 3.80)	0,174	-3.37, 6.49	0	0,641	NS

Luo et al.	2022	Ketogenic Diet	FPI obese	pmol/L	6	190	WMD	-9.36 (-24.02, 5.30)	0,211	-57.16, 38.45	87	0,788	NS
Luo et al.	2022	Ketogenic Diet	DBP obese with T2D	mmHg	2	113	WMD	-1.88 (-5.49, 1.74)	0,309	NA	0	NA	NS
Luo et al.	2022	Ketogenic Diet	Creatinine obese	mg/dL	4	236	WMD	-0.03 (-0.09, 0.03)	0,314	-0.67, 0.61	70	0,609	NS
Luo et al	2022	Ketogenic Diet	BW obese	kg	9	397	WMD	-0.98 (-3.22, 1.27)	0,395	-3.69, 1.74	0	0,037	NS
Luo et al	2022	Ketogenic Diet	FPI obese with T2D	pmol/L	3	145	WMD	-3.86 (-12.92, 5.20)	0,403	-66.30, 58.57	3	0,784	NS
Luo et al.	2022	Ketogenic Diet	DBP obese	mmHg	4	293	WMD	0.88 (-1.22, 2.98)	0,412	-4.06, 5.81	3	0,423	NS
Luo et al.	2022	Ketogenic Diet	Uric Acid obese	mg/dL	4	156	WMD	0.67 (-1.19, 2.53)	0,481	-23.36, 24.69	100	0,936	NS
Luo et al.	2022	Ketogenic Diet	HbA1c obese	%	4	269	WMD	-0.04 (-0.20, 0.11)	0,574	-0.63, 0.54	52	0,774	NS
Luo et al.	2022	Ketogenic Diet	WC obese	cm	4	229	WMD	-0.74 (-3.64, 2.16)	0,615	-10.68, 9.20	35	0,865	NS
Luo et al.	2022	Ketogenic Diet	SBP obese with T2D	mmHg	2	113	WMD	1.69 (-5.62, 9.00)	0,650	NA	0	NA	NS
Luo et al.	2022	Ketogenic Diet	HOMA obese	.	3	117	WMD	0.19 (-0.77, 1.15)	0,696	-10.70, 11.09	69	0,860	NS
Luo et al	2022	Ketogenic Diet	Urea Nitrogen obese	mg/dL	4	235	WMD	0.48 (-17.36, 18.33)	0,958	-84.54, 85.51	95	0,524	NS

Espinoza et al.	2021	Ketogenic Diet	TG	-	8	943	SMD	0.04 (-0.11, 0.19)	0,582	-0.14, 0.23	0	0,000	NS
Espinoza et al.	2021	Ketogenic Diet	HDL-c	-	8	943	SMD	0.04 (-0.11, 0.19)	0,588	-0.15, 0.23	0	0,000	NS
Espinoza et al.	2021	Ketogenic Diet	LDL-c	-	8	943	SMD	0.04 (-0.11, 0.19)	0,590	-0.15, 0.23	0	0,000	NS
Espinoza et al.	2021	Ketogenic Diet	TC	-	8	943	SMD	0.03 (-0.12, 0.19)	0,672	-0.16, 0.23	0	0,511	NS
Espinoza et al.	2021	Ketogenic Diet	BMI	-	3	943	SMD	0.04 (-0.24, 0.32)	0,774	-1.79, 1.87	0	0,171	NS
Smith et al.	2020	Ketogenic Diet	BW (ketogenic)	kg	5	3340	MD	-1.69 (-4.71, 1.33)	0,272	-12.53, 9.15	79	0,900	NS
HP Diet													
Schwingshackl & Hoffmann	2013	HP Diet	HDL-c	mg/dL	11	1276	MD	1.50 (0.37, 2.62)	0,009	0.20, 2.80	0	0,166	Weak
Schwingshackl & Hoffmann	2013	HP Diet	FPI	pmol/L	11	1201	MD	-11.56 (-21.24, -1.87)	0,019	-22.74, -0.38	0	0,841	Weak
Zhao et al.	2018	HP Diet	TG	-	14	694	SMD	-0.20 (-0.35, -0.05)	0,01	-0.36, -0.03	0	0,232	Weak
Zhao et al	2018	HP Diet	TC	-	14	915	SMD	-0.13 (-0.27, 0.02)	0,091	-0.40, 0.14	12	0,145	NS
Zhao et al.	2018	HP Diet	BW	-	16	1162	SMD	-0.08 (-0.21, 0.04)	0,171	-0.22, 0.05	0	0,532	NS

Zhao et al.	2018	HP Diet	DBP	-	11	787	SMD	-0.10 (-0.27, 0.06)	0,207	-0.40, 0.19	14	0,081	NS
Zhao et al.	2018	HP Diet	HbA1c	-	13	933	SMD	-0.07 (-0.20, 0.06)	0,261	-0.22, 0.07	0	0,764	NS
Zhao et al	2018	HP Diet	SBP	-	12	875	SMD	-0.08 (-0.21, 0.06)	0,268	-0.23, 0.08	0	0,003	NS
Zhao et al.	2018	HP Diet	FPG	-	13	856	SMD	-0.09 (-0.26, 0.08)	0,305	-0.46, 0.28	23	0,585	NS
Zhao et al	2018	HP Diet	LDL-c	-	14	915	SMD	-0.06 (-0.19, 0.07)	0,377	-0.20, 0.09	0	0,159	NS
Zhao et al.	2018	HP Diet	BMI	-	8	490	SMD	-0.06 (-0.24, 0.12)	0,492	-0.28, 0.16	0	0,403	NS
Zhao et al.	2018	HP Diet	HDL-c	-	14	917	SMD	-0.04 (-0.22, 0.13)	0,617	-0.46, 0.37	27	0,634	NS
Zhao et al.	2018	HP Diet	FPI	-	9	370	SMD	-0.04 (-0.24, 0.17)	0,721	-0.29, 0.21	0	0,317	NS
Zhao et al.	2018	HP Diet	Fat-free Mass	-	7	274	SMD	-0.04 (-0.27, 0.20)	0,765	-0.35, 0.28	0	0,716	NS
Zhao et al.	2018	HP Diet	Fat Mass	-	8	568	SMD	-0.01 (-0.18, 0.15)	0,884	-0.22, 0.19	0	0,117	NS
Ajala et al.	2013	HP Diet	HbA1c	%	2	137	MD	-0.19 (-0.50, 0.12)	0,230	-	1	NA	NS
DASH Diet													

Lari et al	2021	DASH	SBP	mmHg	37	6011	MD	-3.94 (-5.24, -2.64)	≤0,001	-9.41, 1.53	76	0,106	Highly Suggestive
Lari et al.	2021	DASH	DBP	mmHg	37	6011	MD	-2.44 (-3.44, -1.45)	≤0,001	-6.76, 1.87	81	0,005	Suggestive
Lari et al.	2021	DASH	BW	kg	27	2755	MD	-1.59 (-2.27, -0.90)	≤0,001	-4.14, 0.96	63	0,686	Suggestive
Lari et al.	2021	DASH	BMI	kg/m2	22	2526	MD	-0.63 (-0.92, -0.35)	≤0,001	-1.65, 0.38	73	0,689	Suggestive
Lari et al.	2021	DASH	WC	cm	16	1722	MD	-1.93 (-2.80, -1.07)	≤0,001	-4.52, 0.66	57	0,070	Suggestive
Lari et al	2021	DASH	TC	mg/dL	25	4834	MD	-5.12 (-8.88, -1.35)	0,008	-20.56, 10.33	73	0,076	Weak
Lari et al.	2021	DASH	LDL-c	mg/dL	25	4953	MD	-3.54 (-6.93, -0.15)	0,041	-18.43, 11.35	76	0,075	Weak
Ndanuko et al.	2016	DASH	SBP	mmHg	11	1957	MD	-4.90 (-6.22, -3.58)	≤0,001	-9.01, -0.78	70	0,332	Weak
Ndanuko et al.	2016	DASH	DBP	mmHg	11	1957	MD	-2.63 (-3.34, -1.91)	≤0,001	-4.73, -0.52	61	0,142	Weak
Lari et al	2021	DASH	VLDL-c	mg/dL	7	373	MD	-2.16 (-4.42, 0.11)	0,062	-7.59, 3.28	35	0,918	NS
Lari et al.	2021	DASH	TG	mg/dL	26	3477	MD	-4.22 (-8.73, 0.30)	0,067	-17.27, 8.83	30	0,312	NS
Lari et al.	2021	DASH	HOMA-IR	-	9	1364	MD	-0.15 (-0.35, 0.05)	0,142	-0.69, 0.39	72	0,012	NS

Lari et al.	2021	DASH	CRP	mg/dL	6	553	MD	-0.03 (-0.08, 0.01)	0,174	-0.18, 0.11	73	0,259	NS
Lari et al.	2021	DASH	HDL-c	mg/dL	28	5112	MD	0.32 (-0.56, 1.20)	0,475	-3.04, 3.68	59	0,466	NS
Lari et al.	2021	DASH	FPI	pmol/L	13	1643	MD	-0.56 (-4.82, 3.71)	0,798	-9.94, 8.83	64	0,059	NS
Lari et al.	2021	DASH	FPG	mg/dL	19	2030	MD	-0.02 (-0.38, 0.35)	0,935	-0.75, 0.72	32	0,033	NS
	Portfolio Diet												
Chiavaroli et al	2018	Portfolio Diet	LDL-c	-	7	604	SMD	-13.05 (-16.04, -10.06)	≤0,001	-22.01, -4.09	66	0,006	Highly Suggestive
Chiavaroli et al.	2018	Portfolio Diet	Non-HDL-c	-	7	604	SMD	-14.99 (-18.43, -11.55)	≤0,001	-24.92, -5.06	60	0,018	Highly Suggestive
Chiavaroli et al.	2018	Portfolio Diet	ApoB	-	7	604	SMD	-18.13 (-22.74, -13.51)	≤0,001	-30.99, -5.27	51	0,091	Highly Suggestive
Chiavaroli et al.	2018	Portfolio Diet	TC	-	7	604	SMD	-13.64 (-19.94, -7.33)	≤0,001	-35.49, 8.22	90	0,052	Suggestive
Chiavaroli et al.	2018	Portfolio Diet	TG	-	7	604	SMD	-5.04 (-7.51, -2.58)	≤0,001	-11.65, 1.57	57	0,636	Suggestive
Chiavaroli et al.	2018	Portfolio Diet	CHD risk	-	7	604	SMD	-1.34 (-2.19, -0.49)	0,002	-3.89, 1.20	54	0,312	Weak
Chiavaroli et al.	2018	Portfolio Diet	DBP	--	7	604	SMD	-1.36 (-2.33, -0.38)	0,006	-2.63, -0.08	0	0,089	Weak

Chiavaroli et al.	2018	Portfolio Diet	CRP	-	7	604	SMD	-0.55 (-0.97, -0.12)	0,012	-1.46, 0.37	24	0,613	Weak
Chiavaroli et al.	2018	Portfolio Diet	SBP	-	7	604	SMD	-1.75 (-3.23, -0.26)	0,021	-3.69, 0.20	0	0,402	Weak
Chiavaroli et al.	2018	Portfolio Diet	HDL-c	-	7	604	SMD	-0.27 (-0.94, 0.40)	0,422	-1.75, 1.20	27	0,839	NS
Chiavaroli et al.	2018	Portfolio Diet	BW	-	7	604	SMD	-0.10 (-0.48, 0.27)	0,591	-0.60, 0.39	0	0,906	NS
Nordic Diet													
Massara et al.	2022	Nordic Diet	Stroke Incidence (extreme quintiles)	%	4	124152	MD	0.87 (0.78, 0.96)	≤0,001	0.68, 1.07	I2	0,148	Highly Suggestive
Massara et al.	2022	Nordic Diet	T2D (extreme quintiles)	%	6	112157	MD	0.95 (0.85, 1.05)	≤0,001	0.67, 1.23	0	0,221	Highly Suggestive
Massara et al.	2022	Nordic Diet	CVD Mortality (extreme quintiles)	%	7	637049	MD	0.80 (0.70, 0.90)	≤0,001	0.56, 1.04	49	0,815	Highly Suggestive
Massara et al.	2022	Nordic Diet	CHD Incidence (extreme quintiles)	%	5	123382	MD	0.83 (0.64, 1.02)	≤0,001	0.20, 1.46	45	0,472	Suggestive
Massara et al.	2022	Nordic Diet	CVD Incidence (extreme quintiles)	%	3	60436	MD	0.80 (0.59, 1.02)	≤0,001	-1.90, 3.51	67	0,170	Suggestive
Massara et al.	2022	Nordic Diet	BMI	kg/m2	4	429	MD	-0.94 (-1.26, -0.61)	≤0,001	-1.95, 0.08	91	0,506	Weak

Massara et al.	2022	Nordic Diet	FPI	pmol/L	4	478	MD	-7.83 (-12.26, -3.39)	0,001	-17.56, 1.91	19	0,565	Weak
Massara et al.	2022	Nordic Diet	BW	kg	6	744	MD	-2.00 (-3.24, -0.75)	0,002	-6.22, 2.23	90	0,411	Weak
Massara et al.	2022	Nordic Diet	DBP	mmHg	4	542	MD	-1.55 (-2.77, -0.33)	0,013	-5.70, 2.59	88	0,282	Weak
Massara et al.	2022	Nordic Diet	WC	cm	4	492	MD	-1.71 (-3.13, -0.29)	0,018	-7.29, 3.88	35	0,578	Weak
Ramezani-Jolfaie et al.	2018	Nordic Diet	TC	mg/dL	5	513	WMD	-6.93 (-13.68, -0.19)	0,044	-32.54, 18.67	57	0,661	Weak
Ramezani-Jolfaie et al.	2018	Nordic Diet	HDL-c	mg/dL	10	1026	WMD	-2.90 (-4.65, -1.16)	0,001	-9.04, 3.24	0	0,084	Weak
Ndanuko et al.	2016	Nordic Diet	SBP	mmHg	3	1957	MD	-5.20 (-7.30, -3.11)	≤0,001	-18.79, 8.38	0	0,752	Weak
Ndanuko et al.	2016	Nordic Diet	DBP	mmHg	3	1957	MD	-3.85 (-5.50, -2.19)	≤0,001	-14.57, 6.87	0	0,627	Weak
Massara et al.	2022	Nordic Diet	HDL-c	mg/dL	5	644	MD	-0.95 (-2.07, 0.16)	0,094	-4.63, 2.73	67	0,598	NS
Massara et al.	2022	Nordic Diet	LDL-c	mg/dL	5	617	MD	-3.65 (-8.39, 1.08)	0,130	-21.60, 14.29	93	0,356	NS
Massara et al.	2022	Nordic Diet	non-HDL-c	mg/dL	4	497	MD	-4.02 (-9.62, 1.57)	0,159	-16.31, 8.26	0	0,486	NS
Massara et al.	2022	Nordic Diet	ApoB	mg/dL	2	252	MD	-14.50 (-35.08, 6.08)	0,167	NA	1	NA	NS

Massara et al.	2022	Nordic Diet	TG	mg/dL	5	644	MD	-0.85 (-2.54, 0.85)	0,326	-5.69, 4.00	43	0,048	NS
Massara et al.	2022	Nordic Diet	CRP	mg/dL	5	644	MD	-0.85 (-2.94, 1.24)	0,427	-7.07, 5.38	65	0,493	NS
Massara et al.	2022	Nordic Diet	FPG	mg/dL	5	644	MD	-0.34 (-1.54, 0.86)	0,584	-3.83, 3.15	47	0,118	NS
Massara et al.	2022	Nordic Diet	SBP	mmHg	4	542	MD	-0.94 (-4.81, 2.92)	0,633	-18.03, 16.15	78	0,117	NS
Ramezani-Jolfaie et al.	2018	Nordic Diet	TG	mg/dL	5	513	WMD	-0.13 (-2.37, 2.11)	0,909	-7.22, 6.96	59	0,261	NS
Vegetarian- Vegan Diet													
Wang et al.	2015	Vegetarian vs. omnivorous Diet	HDL-c	mg/dL	9	641	WMD	-1.84 (-2.41, -1.28)	≤0,001	-2.52, -1.16	0	0,999	Highly Suggestive
Wang et al.	2015	Vegetarian Diet	TC	mg/dL	10	774	WMD	-6.64 (-9.96, -3.33)	≤0,001	-16.17, 2.88	56	0,397	Suggestive
Termannsen et al.	2022	Vegan Diet	BW (no Dietary interventions)	kg	3	322	MD	-7.43 (-10.20, -4.65)	≤0,001	-40.14, 25.29	79	0,461	Weak
Termannsen et al.	2022	Vegan Diet	BMI (no Dietary interventions)	kg/m2	3	322	MD	-2.78 (-3.91, -1.65)	≤0,001	-15.79, 10.24	72	0,684	Weak
Termannsen et al.	2022	Vegan Diet	HbA1c (BMI>26)	%	3	366	MD	-0.08 (-0.11, -0.04)	≤0,001	-0.31, 0.16	4	0,920	Weak
Termannsen et al.	2022	Vegan Diet	HbA1c (T2DM)	%	6	321	MD	-0.38 (-0.55, -0.20)	≤0,001	-0.69, -0.06	10	0,637	Weak

Termannsen et al.	2022	Vegan Diet	BMI (other Dietary interventions)	kg/m2	7	458	MD	-0.87 (-1.34, -0.40)	≤0,001	-2.42, 0.68	82	0,346	Weak
Termannsen et al.	2022	Vegan Diet	BW (other Dietary interventions)	kg	7	375	MD	-2.72 (-4.23, -1.21)	≤0,001	-7.68, 2.24	80	0,469	Weak
Termannsen et al.	2022	Vegan Diet	LDL-c	mg/dL	8	687	MD	-4.40 (-7.28, -1.51)	0,003	-12.63, 3.84	55	0,811	Weak
Termannsen et al.	2022	Vegan Diet	TC	mg/dL	8	605	MD	-5.48 (-9.42, -1.54)	0,006	-17.38, 6.41	65	0,274	Weak
Termannsen et al.	2022	Vegan Diet	HDL-c	mg/dL	9	698	MD	-1.19 (-2.21, -0.17)	0,023	-3.96, 1.59	53	0,515	Weak
Wang et al.	2015	Vegetarian vs. omnivorous Diet	non-HDL-c	mg/dL	8	583	WMD	-5.04 (-8.58, -1.49)	0,005	-15.03, 4.95	57	0,403	Weak
Wang et al.	2015	Vegetarian vs. omnivorous Diet	LDL-c	mg/dL	7	572	WMD	-5.22 (-9.48, -0.96)	0,016	-18.53, 8.10	75	0,812	Weak
Termannsen et al.	2022	Vegan Diet	TG	mg/dL	9	668	MD	1.90 (-1.51, 5.31)	0,274	-8.23, 12.04	64	0,767	NS
Termannsen et al. (	2022	Vegan Diet	SBP	mmHg	8	466	MD	1.28 (-1.54, 4.11)	0,374	-5.26, 7.82	34	0,348	NS
Termannsen et al	2022	Vegan Diet	DBP	mg/dL	11	832	MD	0.54 (-1.21, 2.29)	0,545	-3.64, 4.72	37	0,486	NS
Wang et al	2015	Vegetarian vs. omnivorous Diet	TG	mmHg	8	466	WMD	0.69 (-0.97, 2.36)	0,415	-2.74, 4.12	21	0,173	NS

Table S2. AMSTAR 2 tool for the Quality assessment of included meta-analyses .

Reference	1	2	3	4	5	6	7	8	9	10	11	12	13	14	15	16	Quality Assessment
**Amini et al. (2024)** [25]	YES	NO	YES	PY	NO	YES	YES	YES	YES	NO	YES	YES	YES	YES	YES	YES	**Low**
**Choy & Louie (2023)** [26]	YES	NO	YES	PY	YES	YES	NO	YES	YES	YES	YES	YES	YES	YES	NO	YES	**Critically Low**
**Luo et al (2022)** [27]	YES	NO	YES	PY	YES	YES	YES	YES	YES	YES	YES	YES	YES	NO	YES	YES	**Low**
**Massara et al. (2022)** [24]	YES	YES	YES	PY	NO	YES	NO	YES	YES	YES	YES	YES	NO	YES	YES	YES	**Critically Low**
**Rafiullah et al. (2022)** [28]	YES	YES	YES	PY	NO	NO	YES	YES	YES	YES	YES	YES	NO	YES	YES	YES	**Low**
**Termannsen et al. (2022)** [29]	YES	YES	YES	PY	YES	NO	NO	YES	YES	YES	YES	YES	NO	NO	NO	YES	**Critically Low**
**Chiavaroli et al. (2021)** [30]	NO	YES	YES	PY	YES	YES	NO	YES	YES	YES	YES	YES	NO	NO	YES	YES	**Critically Low**
**Espinoza et al. (2021)** [31]	YES	NO	YES	PY	YES	YES	NO	YES	YES	YES	YES	YES	YES	YES	NO	YES	**Critically Low**
**Lari et al. (2021)** [1]	YES	YES	YES	PY	YES	YES	NO	YES	YES	NO	YES	YES	YES	NO	YES	YES	**Low**
**Smith et al. (2020)** [32]	YES	YES	YES	PY	NO	NO	YES	YES	NO	NO	YES	NO	NO	NO	NO	NO	**Critically Low**
**Ojo et al. (2019)** [5]	YES	NO	YES	PY	NO	NO	NO	YES	YES	YES	YES	NO	NO	YES	NO	YES	**Critically Low**
**Ramezani-Jolfaie et al. (2019)** [33]	YES	YES	YES	PY	NO	YES	YES	YES	YES	YES	YES	YES	NO	YES	YES	YES	**Low**
**Chiavaroli et al. (2018)** [22]	YES	YES	YES	PY	NO	YES	NO	YES	YES	YES	YES	YES	YES	YES	YES	YES	**Low**

**Sainsbury et al. (2018)** [34]	YES	YES	YES	PY	YES	YES	NO	YES	YES	YES	YES	YES	YES	YES	NO	YES	**Critically Low**
**Zhao et al. (2018)** [6]	YES	NO	YES	PY	NO	YES	NO	YES	YES	YES	YES	YES	YES	YES	YES	NO	**Critically Low**
**Meng et al. (2017)** [35]	YES	NO	YES	PY	YES	YES	NO	YES	YES	YES	YES	YES	YES	YES	YES	YES	**Critically Low**
**Ndanuko et al. (2016)** [36]	YES	YES	YES	PY	NO	NO	NO	YES	YES	NO	YES	YES	YES	YES	NO	NO	**Critically Low**
**Nissensohn et al. (2016)** [37]	YES	NO	YES	PY	YES	NO	NO	YES	YES	NO	YES	YES	YES	YES	NO	NO	**Critically Low**
**Wang et al. (2015)** [3]	YES	NO	YES	PY	NO	YES	NO	YES	YES	YES	YES	YES	YES	YES	YES	YES	**Critically Low**
**Huo et al. (2015)** [7]	YES	NO	YES	PY	YES	NO	NO	YES	YES	NO	YES	YES	NO	NO	YES	YES	**Critically Low**
**Ajala et al. (2013)** [23]	YES	NO	YES	PY	NO	NO	YES	YES	YES	NO	YES	NO	NO	NO	NO	NO	**Critically Low**
**Bueno et al. (2013)** [38]	YES	YES	YES	PY	NO	NO	NO	YES	YES	NO	YES	YES	YES	YES	NO	NO	**Critically Low**
**Schwingshackl & Hoffmann (2013)** [39]	YES	YES	YES	PY	NO	NO	NO	YES	YES	NO	YES	YES	YES	YES	YES	NO	**Low**
**Fleming & Codwin (2013)** [40]	YES	YES	YES	PY	YES	NO	NO	YES	YES	YES	YES	YES	NO	NO	NO	YES	**Critically Low**
**Santos et al. (2012)** [41]	YES	NO	YES	PY	NO	NO	NO	YES	YES	NO	YES	YES	NO	YES	NO	YES	**Critically Low**

1. Did the research questions and inclusion criteria for the review include the components of PICO?

2. Did the report of the review contain an explicit statement that the review methods were established prior to the conduct of the review and did the report justify any significant deviations from the protocol?

3. Did the review authors explain their selection of the study designs for inclusion in the review?

4. Did the review authors use a comprehensive literature search strategy?

5. Did the review authors perform study selection in duplicate?

6. Did the review authors perform data extraction in duplicate?

7. Did the review authors provide a list of excluded studies and justify the exclusions?

8. Did the review authors describe the included studies in adequate detail?

9. Did the review authors use a satisfactory technique for assessing the risk of bias (RoB) in individual studies that were included in the review?

10. Did the review authors report on the sources of funding for the studies included in the review?

11. If meta-analysis was performed did the review authors use appropriate methods for statistical combination of results?

12. If meta-analysis was performed, did the review authors assess the potential impact of RoB in individual studies on the results of the meta-analysis or other evidence synthesis?

13. Did the review authors account for RoB in individual studies when interpreting/ discussing the results of the review?

14. Did the review authors provide a satisfactory explanation for, and discussion of, any heterogeneity observed in the results of the review?

15. If they performed quantitative synthesis did the review authors carry out an adequate investigation of publication bias (small study bias) and discuss its likely impact on the results of the review?

16. Did the review authors report any potential sources of conflict of interest, including any funding they received for conducting the review?
